# Nursing staff’s instrument for change-of-shift reporting - SBAR (Situation-Background-Assessment-Recommendation): validation and application

**DOI:** 10.1590/0034-7167-2021-0608

**Published:** 2022-08-22

**Authors:** Tânia Roberta Limeira Felipe, Wilza Carla Spiri, Carmen Maria Casquel Monti Juliani, Maria Eugênia Guerra Mutro

**Affiliations:** I Universidade Estadual Paulista Júlio de Mesquita Filho. Botucatu, São Paulo, Brazil; II Secretaria Municipal de Saúde. Bauru, São Paulo, Brazil

**Keywords:** Validation Studies, Hospital Communication Systems, Nursing Team, Professional-Patient Relations, Nursing Administration Research., Estudios de Validación, Sistemas de Comunicación en Hospital, Grupo de Enfermería, Relaciones Profesional-Paciente, Investigación en Administración de Enfermería., Estudos de Validação, Sistemas de Comunicação no Hospital, Equipe de Enfermagem, Relações Profissional-Paciente, Pesquisa em Administração de Enfermagem.

## Abstract

**Objectives:**

to validate and apply a change-of-shift instrument using the SBAR (Situation-Background-Assessment-Recommendation) tool.

**Methods:**

methodological study for the validation of an instrument. It was validated by ten judges from the area of nursing teaching and care and applied in a surgical gastroenterology ward by 11 nursing technicians in February 2019. The analyses considered descriptive statistics.

**Results:**

the judges analyzed the instrument with a content validity index of 91.7% and made suggestions, which led to the second version of the instrument. The participants reported that the predominant modality of shift handover is oral, in the nursing room, lasting six to ten minutes. Most pay attention during shift change, mention that delays and early departures interfere in the activity and believe that the instrument provides the necessary information and is viable.

**Conclusions:**

the instrument built was validated, and its application proved relevant, as it was considered necessary and feasible.

## RESUMO

**Objetivos:**

validar e aplicar instrumento de passagem de plantão utilizando a ferramenta SBAR *(Situation-Background-Assessment-Recommendation).*

**Métodos:**

estudo metodológico para a validação de instrumento. O instrumento foi validado por dez juízes da área de ensino e assistência de enfermagem e aplicado em uma enfermaria de gastroenterologia cirúrgica por 11 técnicos de enfermagem no mês de fevereiro de 2019. As análises consideraram a estatística descritiva.

**Resultados:**

os juízes analisaram o instrumento com índice de validade de conteúdo de 91,7%, fizeram sugestões gerando a segunda versão do instrumento. Os participantes referiram que a modalidade de passagem de plantão predominante é oral, na sala de enfermagem, de 6 a 10 minutos. A maioria presta atenção na passagem de plantão, refere que atrasos e saídas antecipadas interferem, acreditam que o instrumento possui informações necessárias e é viável.

**Conclusões:**

o instrumento construído foi validado e sua aplicação evidenciou a relevância, pois considera-se o instrumento necessário e viável.

## RESUMEN

**Objetivos:**

validar y aplicar instrumento del pasaje de plantón utilizando la herramienta SBAR (*Situation-Background-Assessment-Recommendation*).

**Métodos:**

estudio metodológico para la validación de instrumento. Este fue validado por diez jueces del área de enseñanza y asistencia de enfermería y aplicado en una enfermería de gastroenterología quirúrgica por 11 técnicos de enfermería en febrero de 2019. Los análisis consideraron la estadística descriptiva.

**Resultados:**

los jueces analizaron el instrumento con índice de validez de contenido de 91,7%, hicieron sugestiones generando la segunda versión del instrumento. Los participantes refirieron que la modalidad del pasaje de plantón predominante es oral, en la sala de enfermería, de seis a diez minutos. La mayoría presta atención en el pasaje de plantón, refiere que retrasos y salidas anticipadas interfieren, creen que el instrumento posee informaciones necesarias y es viable.

**Conclusiones:**

el instrumento construido fue validado, y su aplicación evidenció la relevancia, pues es considerado necesario y viable.

## INTRODUCTION

Change-of-shift reporting is considered a communication process with specific patient information, which is passed from one health professional to another, from one team of care professionals to another, or from health professionals to patients and family members when they go home^(1)^.

The World Health Organization has developed strategies that should be considered during shift handover, with an emphasis on four aspects: 1) ensuring that the healthcare organization implements, in a standardized manner, communication via the use of the SBAR (Situation Background-Assessment-Recommendation) technique during shift change. Consider allocating sufficient time for important information to be communicated, without interruptions. Information regarding the patient’s condition, medications, treatment plans, and changes in the patient’s condition are essential; 2) safeguard that healthcare organizations implement systems to ensure that patients are discharged with all information necessary for their treatment, such as diagnosis, treatment plan, medications, and test results; 3) incorporate training for communication during shift-changes on an ongoing basis; 4) encourage communication between the health care organization and care providers (formal and informal)^(1)^.

The SBAR tool consists of quick and standardized questioning, evaluating four criteria, so that everyone shares precise and focused information, reducing the need for repetition and allowing the elaboration of detailed information^(2)^.

It is a communication tool recommended by Joint Commission International and adopted in many international health services^(3)^. Through it, it is possible to develop critical thinking and consolidate communication skills. Critical thinking involves thinking logically to solve problems, and one of the prerequisites is to apply this tool in the context of practice^(3)^. The SBAR tool makes it possible to structure communication among the healthcare team, especially the nursing team, in an organized, clear and objective manner.

The use of this tool allows communication errors to decrease and contributory factors that improve safety attitudes to be increased, since it is a standardized form of shift change valid for communication among the health team^(4)^. Structured communication techniques such as SBAR improve the perception among healthcare team members, the process of change-of-shift reporting and the collaboration required for this^(5)^.

In the context of adopting the nursing team’s tool for change-of-shift reporting, it is important to mention that in “Situation” a concise report of the patient’s condition is structured. In “Background”, pertinent information about the patient’s case is reported, such as previous history, diagnostic hypothesis, among others. In “Assessment” the patient’s clinical case is stratified, providing real data to support decision making. Finally, in “recommendation”, the nurse recommends actions to the nursing team by analyzing the patient’s needs^(2)^.

Studies conducted in the United States of America showed that the use of the SBAR tool for changing shifts of the nursing team promoted better structure, consistency, prioritization, accuracy, and understanding of the information necessary for care. In addition, the use of the technique provided better communication and knowledge about the assisted patients^(2,6)^.

It stands out as a tool used in the communication process in an expanded manner among the health team. However, this study focuses on the nursing teams’ shift changes, as this is a fragile aspect of nursing care, since, in most experiences, it is performed in an unsystematic way^(6)^.

Although its use is consolidated by nurses in North American and European countries, there is little literature in Brazil on the use of this tool, especially for the shift change of nursing professionals. A study conducted in Brazil concluded that this process, in the hospital context, is carried out empirically, with a lack of tools for its quality, highlighting the scarcity of studies on the SBAR model in the reality of the shift changes in this context^(7)^.

Thus, for establishing this tool, in a first stage, an instrument was built for change-of-shift reporting in the gastroenterology surgical ward of a teaching hospital in the state of São Paulo, based on the needs of this ward and on the literature review on the items required for the SBAR tool^(8)^.

Therefore, this study aims to continue the construction stage of this instrument for its validation and implementation.

## OBJECTIVES

To validate and apply a nursing change-of-shift reporting instrument using the SBAR (Situation Background-Assessment-Recommendation) tool.

## METHODS

### Ethical aspects

The study was developed after approval by the Research Ethics Committee. Data was provided voluntarily by the participants who agreed to the study by signing the Informed Consent Form.

### Study design, period, and location

Methodological study whose objective is to work with complex instruments and tools and to develop methodological references^(9)^. The approach was quantitative and cross-sectional.

Initially, an instrument for shift changes in the surgical gastroenterology ward of a teaching hospital in the state of São Paulo was built using the SBAR tool, considering patient identification data; indicators; Situation (S) (day of admission, medical diagnosis, nursing diagnoses or reports of nursing problems in the last 24 hours); Background (B) (allergies, comorbidities, surgical history, isolation/precautions and communication barriers); Assessment (A) (vital signs, oxygenation/ventilation, consciousness, mobility, drains, catheters, probes, exams, nutritional aspects, dressings, eliminations, medications, and complications); Recommendation (R) (interconsultations, nursing interventions, and other necessary data)^(8)^.

The instrument was applied during the 28 days of February 2019.

### Population, inclusion and exclusion criteria

The study was conducted with judges who were experts in the area of teaching and assistance. In the area of teaching, inclusion occurred through analysis of the curriculum on the Lattes Platform (teachers with expertise in the “medical and surgical nursing” area) and with practical experience in the research’s ward-scenario. Potentially, eight professors would be included in the study, but four responded affirmatively and composed the group of judges with teaching expertise. In assistance, the inclusion criterion was the link of working at the surgical gastroenterology ward, characterizing six judges. All of them agreed to participate in the study. Thus, for the validation of the instrument built^(8)^, the participants were ten judges, divided into two groups: the first group was composed of six nurses with professional experience in clinical practice (surgical gastroenterology), and the second group was represented by four nursing teachers with teaching experience in surgical gastroenterology, the research setting. Therefore, a convenience sample.

To apply the instrument at shift change, after its validation by the judges, the participants were 11 nursing technicians who are part of the staff of the surgical gastroenterology ward of the hospital-research setting. Nurses were not included in this step because they participated in the instrument validation as judges.

### Research Setting

The instrument was applied in a surgical gastroenterology ward of a teaching hospital, with 28 beds. The nursing team consisted of seven nurses, 18 nursing technicians, and three nursing assistants.

At the time of the study, the change-of-shift reporting process among the nursing team members was described considering the involvement of two teams: the one ending their 12-hour shift and the one starting a new one, lasting the same period; the meeting format, with the information considered relevant by the team, was noted in a “draft” document, made useless soon after shift change, and occurred mostly orally, therefore, in an nonsystematic way.

### Procedures for data collection and analysis

The instrument^(8)^ was sent to the judges for validation by means of a form that analyzed the clarity (attribute of what is intelligible, easily understood); pertinence (characteristic of what is appropriate and relevant), and appearance (aspect or that which is shown superficially or at first sight).

For instrument validation, a Content Validity Index (CVI) greater than or equal to 80% was considered, which is the minimum value recommended by the literature^(10)^.

The Delphi technique^(11)^ was used, as it is intended to deduce and refine the opinions of experts in order to reach consensus on a given theme.

The participants who applied the instrument at shift change received it printed in a brochure format for the data collection period (month of February 2019) and were instructed to fill it out during the daily shift change. After the 28 days of use, the participants evaluated the constructed instrument by answering a questionnaire containing sociodemographic data and data regarding the change-of-shift reporting process.

The data were entered into a spreadsheet, and the analysis was performed using descriptive statistics.

## RESULTS

The judges were predominantly female (8; 80%), with professional experience in teaching (4; 40%); had a postgraduate degree (6; 60%); mean age of 42.5 years, and 16.1 years of professional experience.

The CVI was equal to 91.7%, highlighting that the items “Identification, situation, and background clarity”, “Indicators pertinence”, and “Situation and background appearance” had 100% agreement. Table 1 shows all the items evaluated and their agreement.

**Table 1:** Percentage of adequacy of each item’s properties: clarity, pertinence, and appearance, Botucatu, São Paulo, Brazil, 2019

Item	n	%
Identification clarity	10	100
Identification pertinence	9	90
Identification appearance	8	80
Indicator clarity	8	80
Indicator pertinence	10	100
Indicator appearance	9	90
Situation clarity	10	100
Situation pertinence	9	90
Situation appearance	10	100
Background clarity	10	100
Background pertinence	9	90
Background appearance	10	100
Assessment clarity	8	80
Assessment pertinence	9	90
Assessment appearance	8	80
Recommendation clarity	9	90
Recommendation pertinence	9	90
Recommendation appearance	9	90
Mean		91.1

Although the CVI reached an average greater than 80% in the first round, the judges made improvement suggestions on the items proposed in the instrument. The most frequent suggestions (26.8%) referred to the item “Assessment”. These suggestions are described in Table 2.

**Table 2:** Distribution of suggestions per item evaluated, Botucatu, São Paulo, Brazil, 2019

Item mentioned for improvement	n	%
Assessment	11	26.8
Background	7	17.0
Identification	6	14.6
Indicators	6	14.6
Recommendations	6	14.6
Situation	5	12.2
Total	41	100

**Chart 1:**
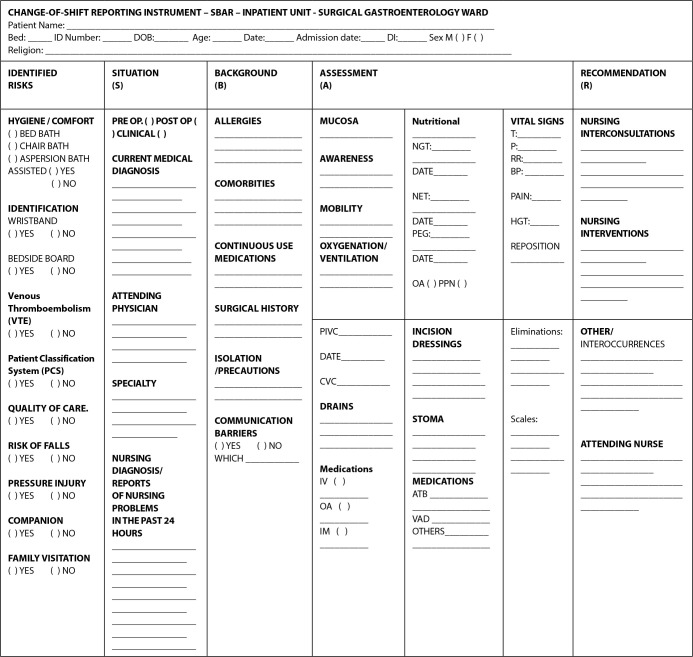
Situation-Background-Assessment-Recommendation instrument for change-of-shift reporting, Botucatu, São Paulo, Brazil, 2019

Thus, changes deemed relevant were updated in the instrument. After modification, the item “Identification” included the registration number, day of hospitalization, and religion; the item “Indicators” was replaced with “Identified risks”, and in this item were included: hygiene and comfort; type of care with the alternatives: bed bath, chair bath, assisted and unaided spray bath; patient classification system, quality of care, and family visits. In all these inclusions, the alternatives for filling in the gaps were “yes” or “no”. For the Situation (S) item, the medical specialty responsible for the patient, the patient’s condition (preoperative or postoperative), and current medical diagnosis were included. For Background (B), nothing was suggested. For Assessment (A), the field “medications” included the most used means of administration, special control drugs, pain scale, and dates of drain and probe changes. In this item, “laboratory tests” were excluded. For the Recommendation (R) item, “interconsultations” was replaced by “nursing interconsultations and intercurrences” (Chart 1).

After the judges’ suggestions were included, the instrument was returned to them for further comments. All reported that the instrument’s appearance, clarity, and relevance were adequate and that it could be applied.

The participants applied 351 change-of-shift reporting instruments. They were predominantly female (10; 90.9%) and with a technical education (9; 81.8%). It is worth noting that, although nursing technician requires only a technical level of education, two of them had a college degree. Table 3 describes this characterization.

**Table 3:** Characteristics of the participants in the application of the change-of-shift reporting instrument (Situation-Background-Assessment-Recommendation) (n = 11), Botucatu, São Paulo, Brazil, 2019

Variable	n	%
Sex Female Male	10 1	90.9 9.1
Age	Mean 37.9 years	
Professional Experience	Mean 10.3 years	
Training Technical level Higher Level	9 2	81.8 18.2

The participants answered the change-of-shift and SBAR tool questionnaire. It is noteworthy that the predominant modality of shift change was oral (7; 63.6%); the location for this was the nursing room (11; 100%); the estimated time was from six to ten minutes for all the patients under the professional’s care responsibility (8; 72.7%); the process being recorded in the book or patient’s chart and not using this moment to clarify doubts was mentioned by six (54.5%) participants; ten (90.9%) informed that their colleague pays attention during the shift change; however, they mentioned there are early departures and delays that interfere in this process (8; 72.7%) and that there are side conversations (10; 90.9%).

All 11 participants considered that the SBAR tool used contains the necessary information, and nine (81.8%) evaluated its use as good and very good for shift change, being feasible for implementation in the unit. As for the information required in this procedure, intercurrences were mentioned by 11 (100%) participants. Table 4 shows this assessment.

**Table 4:** Data regarding change-of-shift reporting and use of the Situation-Background-Assessment-Recommendation tool, Botucatu, São Paulo, Brazil, 2019

Variable	n	%
Change-of-Shift Reporting Format Oral Written Oral and written	7 0 4	63.6 0.0 36.4
Location where the shift change occurs By the patient Unit’s corridor Nursing room Other	0 0 11 0	0.0 0.0 100.0 0.0
Time dedicated to shift handover Up to 5 minutes 6 to 10 minutes 11 to 20 minutes 21 to 30 minutes Above 30 minutes	2 8 1 0 0	18.2 72.7 9.1 0.0 0.0
Documenting of information in book/chart Yes No	6 5	54.5 45.5
Moment for clarifying doubts during handover Yes No	5 6	45.5 54.5
Colleague’s behavior during shift change Pays attention Side conversations Performs procedures	10 0 7	90.9 0.0 63.6
Delays, early departures interfering with shift change Yes No	8 3	72.7 27.3
Interferences during shift change Interruptions Side conversations Noises Questionings Need to repeat information Others	6 10 3 1 3 0	54.5 90.9 27.3 9.1 27.3 0.0
SBAR contains the necessary information Yes No	11 0	100.0 0.0
SBAR Evaluation for change-of-shift reporting Bad Regular Good Very Good Excellent	0 2 6 3 0	0.0 18.2 54.5 27.3 0.0
Necessary information for reporting during shift change Intercurrences Clinical conditions Administrative Matters Exams Medications Changes in treatment Patient identification Family and Companions Care and procedures Others	11 9 0 9 10 7 7 4 8 0	100.0 81.2 0.0 81.2 90.9 63.6 63.6 36.4 72.7 0.0
Feasibility of SBAR implementation in the unit Yes No	9 2	81.8 18.2

*SBAR - Situation-Background-Assessment-Recommendation.*

## DISCUSSION

The instrument was evaluated by ten judges, expert nurses experienced in clinical practice. The selection of the judges considered their experience and qualification. The selection of experts for the evaluation of instruments is as important as the definition of the domains of the instrument, as a systematized method of judgment of information, used to obtain consensus of experts on a given theme aiming at its validation, is what is proposed with the Delphi technique^(11)^.

After the judges’ analysis and even obtaining a 91.7% CVI, there were suggestions for improvement; and, after being analyzed, some items that were in accordance with the SBAR tool proposal were changed. The inclusion and exclusion of items allowed the construction of the second version of the instrument to be applied during the surgical gastroenterology ward’s nurses’ shift handover and analyzed by the nursing technicians of this ward. The proposed format was considered adequate by the judges, and this converges with research that emphasizes that this modality facilitates clinical reasoning and patient information^(12-13)^.

The items that composed the instrument are in accordance with what is proposed by the World Health Organization (WHO), which emphasizes that standardization is an important communication tool; and the SBAR tool is an adequate format for this communication^(1)^.

After validation considering clarity, relevance, and appearance, the instrument underwent analysis with a sample of 11 participants, nursing technicians who make up the functional staff of the study scenario. Their profile is in accordance with the research carried out at a national level about Brazilian nursing, since, in Brazil, 53% of health professionals are technicians and nursing assistants, young, that is, younger than 35 years old (35%), and mostly female (nine out of ten professionals)^(14)^.

The pilot test was implemented, and the participants applied the instruments during a one-month period. According to the literature, this phase is necessary to verify if the instrument is clear and understandable to the members who will use it^(15-17)^.

The participants referred that change-of-shift reporting is predominantly oral and occurs in the nursing room lasting from six to ten minutes with the colleague paying attention. However, when questioned about the interferences in this procedure, they highlighted colleague delays, early departures, and side conversations, which contradicted the previous statement. It is inferred that the participants answered initially what would be ideal in the change-of-shift reporting process and, later, the truthful scenario. The oral modality of this process is corroborated in a study^(18)^.

Contrary to the present study, the location for shift handover proposed in studies was at bedside, because it is a way to promote safety in health services, enabling patient participation in their care^(12,18-19)^.

As for the time for change-of-shift reporting, the literature shows that 10 to 20 minutes is enough, differently from what was found in the present study. The authors refer that the time spent on this action will determine the quantity and quality of information^(12,18)^. Studies show that nurses complain about the prolonged time spent on change-of-shift reporting and point out that performing this at bedside reduces that time, avoiding overtime as a result of delays in the departure of the nursing team^(19-20)^.

Among the factors that negatively interfere in shift changes, authors point out as the most frequent: interruptions, external noises, lack of punctuality, and side conversations among team members^(12,18)^.

Among the questionnaires, all participants considered that the instrument based on the SBAR methodology had the necessary information for the change-of-shift reporting, and most of them evaluated it as good and very good. They also mentioned that the instrument is adequate for implementation in the unit.

A study aiming to implement a change-of-shift reporting tool using the acronym ISBAR (Identification, Situation, Background, Assessment & Action, Response/Rationale) in an emergency care unit concluded that it is crucial to use a standardized, formal, and systematized tool in order to ensure that the transfer of care is “effective, complete, and objective” for patient and team safety^(21)^.

International research highlights that the use of the SBAR tool for the handover of shifts is an effective way to standardize communication between nursing team members, as well as being beneficial to patients and contributing to team satisfaction and patient safety^(2-7,22)^.

This study shows that, for a successful change-of-shift, it is important to develop a form and a standard operating protocol (SOP) that emphasize the guiding elements of this practice and ensure the quality of the process by including necessary and safe information for the continuity of care. The participation of all those involved in assistance is essential so that the information is consistent and reliable^(23)^.

### Study limitations

The limitation of the study refers to the single scenario of hospital care, the reduced number of professionals who applied the tool, and the exclusion of nurses, since they were judges in the validation of the instrument in the chosen scenario.

### Contributions to the field of Nursing

This study contributed to teaching and research in nursing, describing the validation of an instrument. The positive results found give this tool the full condition to be applied in practice, which will enable effective communication between nursing and healthcare teams, for an interprofessional and collaborative practice.

## CONCLUSIONS

The instrument was validated, allowing a new version that considered the judges’ suggestions and was applied during shift changes in a surgical gastroenterology ward.

The participants emphasized that the SBAR tool used has necessary and consistent information. They rated its use as good and very good for change-of-shift reporting and deemed feasible its implementation for use in the unit.

## SUPPLEMENTARY MATERIAL

The manuscript is from a Master’s dissertation and research data is available at: https://repositorio.unesp.br/handle/11449/186328.

0034-7167-reben-75-06-e20210608-sup01
