# Supplementary material for: Nursing staff’s instrument for change-of-shift reporting - SBAR (Situation-Background-Assessment-Recommendation): validation and application
Source: Rev Bras Enferm. 2022 Aug 22;75(6):e20210608. doi: 10.1590/0034-7167-2021-0608 (PMC9749775; doi:10.1590/0034-7167-2021-0608)
Supplement: 0034-7167-reben-75-06-e20210608-sup01 [file 0034-7167-reben-75-06-e20210608-sup01.pdf]

UNIVERSIDADE ESTADUAL PAULISTA “JÚLIO DE MESQUITA FILHO”

FACULDADE DE MEDICINA DE BOTUCATU

TÂNIA ROBERTA LIMEIRA FELIPE

CONSTRUÇÃO E VALIDAÇÃO SEMÂNTICA DE UM INSTRUMENTO DE PASSAGEM DE PLANTÃO

Orientadora: Profª. Drª. Wilza Carla Spiri

BOTUCATU

2019

TÂNIA ROBERTA LIMEIRA FELIPE

CONSTRUÇÃO E VALIDAÇÃO SEMÂNTICA DE UM INSTRUMENTO DE PASSAGEM DE PLANTÃO

Dissertação apresentada ao programa de Mestrado Profissional em Enfermagem da Faculdade de Medicina de Botucatu – UNESP, para obtenção do título de Mestre em Enfermagem.

Linha de Pesquisa: Gerenciamento de Serviços de Saúde e de Enfermagem.

Orientadora: Prof^a. Dr^a. Wilza Carla Spiri

BOTUCATU

2019

FICHA CATALOGÁFICA

FICHA CATALOGRÁFICA ELABORADA PELA SEÇÃO TÉC. AQUIS. TRATAMENTO DA INFORM.
DIVISÃO TÉCNICA DE BIBLIOTECA E DOCUMENTAÇÃO - CÂMPUS DE BOTUCATU - UNESP
BIBLIOTECÁRIA RESPONSÁVEL: ROSANGELA APARECIDA LOBO-CRB 8/7500

Felipe, Tânia Roberta Limeira.
Construção e validação semântica de um instrumento de passagem de plantão / Tânia Roberta Limeira Felipe. - Botucatu, 2019

Dissertação (mestrado) - Universidade Estadual Paulista "Júlio de Mesquita Filho", Faculdade de Medicina de Botucatu
Orientador: Wilza Carla Spiri
Capes: 40401006

1. Equipe de enfermagem. 2. Estudos de validação. 3. Enfermagem - Prática. 4. Gerenciamento da prática profissional. 5. Comunicação em saúde.

Palavras-chave: Equipe de enfermagem; Estudos de validação; Gerenciamento na prática de enfermagem; Sistema de comunicação no hospital.

FOLHA DE APROVAÇÃO

Nome: Tânia Roberta Limeira Felipe

Título: Construção e validação semântica de um instrumento de passagem de plantão

Dissertação apresentada à Faculdade de Medicina, Universidade "Júlio de Mesquita Filho" Campus de Botucatu, para obtenção do título de Mestre em Enfermagem

Orientadora: Profa. Dra.Wilza Carla Spiri

Aprovado em: ___/___/____

Banca examinadora

Profa. Dra...................................Universidade..............

Julgamento:.................................Assinatura:.................

Profa. Dra...................................Universidade..............

Julgamento:.................................Assinatura:.................

Profa. Dra...................................Universidade..............

Julgamento:.................................Assinatura:.................

Botucatu, 30 de julho de 2019.

Projeto de Pesquisa

2017 - Atual

TECNOLOGIAS DE APOIO À SISTEMATIZAÇÃO DA ASSISTÊNCIA DE ENFERMAGEM: CONTRIBUIÇÕES DE CURSO DE MESTRADO PROFISSIONAL DA REGIÃO CENTRO-SUL PAULISTA

Descrição: Projeto de pesquisa vinculado ao Acordo Capes/Cofen Edital n° 27/2016, cujo o tema é a Sistematização da Assistência de Enfermagem (SAE), apresentando como objeto de estudo, as tecnologias de apoio para sua implementação e avaliação, em diferentes níveis de atenção à saúde, com destaque às relacionadas ao Processo de Enfermagem (PE). O objetivo geral é produzir tecnologias de apoio à SAE/PE, a serem aplicadas em hospitais e rede básica de saúde de municípios da região centro-sul paulista. Especificamente, espera-se qualificar a formação dos discentes do Programa de Pós-graduação em Enfermagem - Curso Mestrado Profissional da Faculdade de Medicina de Botucatu - UNESP (Programa MP), em relação à SAE/PE, ampliar ações de colaboração entre a universidade e os serviços de saúde para a consolidação da SAE/PE e incrementar a produção e divulgação do conhecimento científico e tecnológico sobre SAE/PE. Para tal, após aprovação por Comitê de Ética, serão desenvolvidos dez subprojetos de pesquisa na temática em foco, por discentes do Programa MP. Estes estudos adotarão abordagens qualitativas e/ou quantitativas de pesquisa e realizarão coleta, análise e discussão de dados pertinentes aos respectivos objetivos, com base nos princípios e diretrizes do Sistema Único de Saúde, premissas da SAE/PE, Teoria das Necessidades Humanas Básicas, Teoria do Autocuidado de Orem e dos sistemas de classificação da NANDA-I, NIC, NOC e CIPE. Os contextos institucionais para o desenvolvimento das pesquisas serão os locais de trabalho dos discentes participantes. Como um dos subprojetos consta: Construção e Validação Semântica de um Instrumento de Passagem de Plantão de Tânia Roberta Limeira Felipe, orientação: Profa. Dra. Wilza Carla Spiri (Mestrado).

Situação: Em andamento; Natureza: Pesquisa.

Alunos envolvidos: / Mestrado profissional: (10).

Integrantes: Vera Lucia Pamplona Tonete - Coordenador / Cristina Maria Garcia de Lima Parada - Integrante / Marla Andreia Garcia – Integrante / Marli Terezinha Cassamassimo Duarte - Integrante / Milena Temer Jamas - Integrante / Regina Célia Popim - Integrante / Rodrigo Jensen - Integrante / Silvana Andreia Molina Lima - Integrante / Sílvia Cristina Mangini Bocchi - Integrante / Wilza Carla Spiri - Integrante / Andréia Cristina Yauch – Integrante / Bruna Pegorer Santos – Integrante / Elaine Garcia de Oliveira – Integrante / Jéssica Eugênio Pessan dos Santos – Integrante / Karolline Bertoldo Angelim Avancini – Integrante / Maria Elizandre Camilo de Oliveira – Integrante / Marina Prearo Marina Prearo / Pamela Roustini da Fonseca – Integrante / Rosemary Fermiano – Integrante / Tânia Roberta Limeira Felipe – Integrante
Financiador(es): Coordenação de Aperfeiçoamento de Pessoal de Nível Superior - Cooperação / Conselho Federal de Enfermagem - Auxílio financeiro.
Número de produções C, T & A: 1 / Número de orientações: 1.

DEDICATÓRIA

Dedico...

À minha família, em especial à minha mãe que não mediu esforços para eu chegar nessa etapa da minha vida, sempre me ensinando a agir com respeito, dignidade, honestidade e amor ao próximo.

Aos meus filhos por compreenderem minha ausência e acreditar que os frutos do meu empenho serão colhidos por eles também.

Ao meu pai, que sempre me aconselhou a buscar uma vida melhor através do caminho do conhecimento, sua memória viverá em mim para sempre.

Ao meu irmão que se tornou minha referência em disciplina, lealdade, honestidade e empatia, você é o melhor presente que nossos pais poderiam ter me dado.

E ao meu esposo pelo companheirismo e por torcer pelas minhas escolhas.

AGRADECIMENTOS

Primeiramente à Deus, pela permissão em adentrar por essa porta tão almejada e permitir que pessoas de bem continuem em meu caminho, fazendo sempre o melhor que sabem para a realização dos meus ideais.

À minha orientadora Profª Drª Wilza Carla Spiri que incansavelmente me norteou a esse caminho incrível da pesquisa, com palavras de incentivo e com muita sabedoria, me mostrou quão longe posso chegar. Minha eterna gratidão.

À coordenadora desse curso Profª Dra Vera Lúcia Pamplona Tonete, pelo profissionalismo, pela dedicação aos alunos, pessoas como a senhora transformam o mundo.

Aos professores que pacientemente e sempre prontos me atenderam com muito carinho, permitindo meu crescimento profissional e pessoal.

À Faculdade de Medicina de Botucatu, aos colegas de trabalho, colegas de turma, às enfermeiras Bárbara Priscila Nery dos Santos e Mariele Gobbo, gerente e diretora do Hospital das Clínicas de Botucatu, por mais esse voto de confiança, serei eternamente grata.

Aos enfermeiros e técnicos de enfermagem da enfermaria da Gastroenterologia cirúrgica, porque sem a dedicação deles, também não teria desempenhado um bom trabalho.

Aos funcionários da secretaria da pós-graduação e do departamento de enfermagem, César, Rose e Fernando, por toda paciência que tiveram para que eu pudesse concluir as aulas.

À todas as pessoas que colaboram indiretamente para que possa seguir meu caminho em busca dos meus objetivos.

Em especial...

Ao apoio do Conselho Federal de Enfermagem (COFEn) e da Coordenação de Aperfeiçoamento de Pessoal de Nível Superior (CAPES), por meio do Acordo Capes/Cofen Edital nº 27/2016 - Apoio a Programas de Pós-graduação da Área de Enfermagem - Modalidade Mestrado Profissional, o qual contemplou, com recursos financeiros, o projeto "Tecnologias de apoio à sistematização da assistência de enfermagem: contribuições de curso de mestrado profissional da região centro-sul paulista", do qual o presente estudo faz parte.

EPÍGRAFE

Quero, um dia, dizer às pessoas que nada foi em vão... Que o amor existe, que vale a pena se doar às amizades e às pessoas, que a vida é bela sim e que eu sempre dei o melhor de mim... e que valeu a pena.

Mário Quintana

RESUMO

Introdução: uma das atividades fundamentais, no trabalho de Enfermagem, é a passagem de plantão, a qual é crucial para a garantia da continuidade das ações que estão sendo dispensadas aos pacientes. Nesse sentido, a comunicação torna-se indispensável para que as equipes consigam planejar e executar bem suas atividades, adotando um fluxo adequado de comunicação com canais bem estruturados, facilitando o funcionamento das rotinas assistenciais e administrativas. **Objetivo:** descrever o processo de validação semântica de um instrumento de passagem de plantão utilizando a metodologia SBAR *(Situation-Background-Assessment-Recommendation).* **Método:** trata-se de estudo metodológico para construção e validação de um instrumento de passagem de plantão. Foi realizada uma revisão bibliográfica abrangente sobre a ferramenta SBAR e elaboração do instrumento de passagem de plantão. O instrumento foi validado em março de 2019 por dez juízes da área de ensino e assistência. Posteriormente o instrumento foi aplicado na enfermaria de Gastroenterologia cirúrgica, do hospital cenário da pesquisa no mês de fevereiro de 2019, por 11 técnicos de enfermagem que compõem o quadro funcional dessa enfermaria e que responderam um questionário avaliando o instrumento validado para a passagem de plantão. As análises foram realizadas considerando a estatística descritiva. **Resultados:** os resultados demonstram que os juízes foram constituídos por mulheres (80%), com média de idade de 42,5 anos e tempo de profissão de 16 anos, 40% docentes e 30% de gerentes e assistenciais, com graduação (40%), doutorado (30%), especialização (20%), mestrado e pós-doutorado (10%). Analisaram o instrumento construído segundo a literatura existente sendo que o índice de validade de conteúdo foi de 91,7%. As sugestões pertinentes realizadas pelos juízes foram acatadas. Os participantes foram

técnicos de enfermagem, 90% do sexo feminino. A idade média foi de 37,9 anos e o tempo de profissão com média de 10,3 anos. Destes 18,2% possuem nível superior. A modalidade de passagem de plantão predominante é a oral (63,6%), o local é a sala de enfermagem (100%), o tempo para passagem de plantão é de 6 a 10 minutos, 90,9% prestam atenção na passagem de plantão e 72,7% referem que atrasos e saídas antecipadas interferem na passagem de plantão; 100% referem que o SBAR possui informações necessárias e 81,8% considera ser viável a implantação dessa ferramenta na unidade. A aplicação do instrumento no período de um mês evidenciou que os itens pertencentes ao conjunto de variáveis mais preenchido foram característica do paciente (100%) seguido de situação (90%), riscos identificados e avaliação com 81,8%, breve história (80%) e recomendação (42,7%). **Conclusão**: O instrumento construído foi validado semanticamente e sua aplicação evidenciou a relevância, pois considera-se o instrumento necessário e viável. Os itens com maior preenchimento foram relativos a característica do paciente sendo a identificação o item mais preenchido e o item recomendação teve menor preenchimento, pois como técnicos de enfermagem consideram que alguns itens dessa variável são privativos do enfermeiro.

Descritores: Gerenciamento da prática de enfermagem; Estudos de validação; Sistema de comunicação no hospital; Equipe de enfermagem.

ABSTRACT

Introduction: one of the fundamental activities in nursing work is the change of shift, which is crucial to guarantee the continuity of the actions that are being dispensed to patients. In this sense, communication becomes indispensable for teams to plan and execute their activities, adopting an adequate flow of communication with well-structured channels, facilitating the functioning of care and administrative routines. **Aim**: to describe the process of semantic validation of a change of shift instrument using the SBAR (Situation-Background-Assessment-Recommendation). **Method**: This is a methodological study for the construction and validation of a change of shift instrument. A comprehensive bibliographic review was done on the SBAR tool and preparation of the change of shift instrument. The instrument was validated by ten judges from the academic and assistance area. Afterwards, the instrument was applied to the surgical gastroenterology ward of the research scenario by 11 nursing technicians who compose the functional picture of this ward and who answered a questionnaire evaluating the validated instrument for the change of shift. The analyzes were performed considering the descriptive statistics. **Findings**: the results show that the judges consisted of women (80%), with a average of age of 42.5 years and a profession of 16 years, 40% of professors and 30% of managers and assistants, with graduation (40%), doctorate (30%), specialization (20%), master's degree and post-doctorate (10%). They analyzed the instrument constructed according to the existing literature and the index of content validity was 91.7%. The pertinent suggestions made by the judges were followed. Participants were nursing technicians with 90% female. The mean age was 37.9 years and the time of profession with an average of 10.3 years. Of these, 18.2% have a higher level. The predominant change of shift mode is oral (63.6%), the place is the nursing

office (100%), the time for the change of shift is from 6 to 10 minutes, 90.9% pay attention to the process and 72.7% reported that delays and early departures interfere with the change of shift; 100% report that SBAR has the necessary information and 81.8% believe that it is feasible to implement this tool in the unit. The application of the instrument in the period of one month showed that the items belonging to the most complete set of variables were characteristic of the patient (100%) followed by situation (90%), identified risks and evaluation with 81.8%, brief history (80%) and recommendation (42.7%). **Conclusion**: The built instrument was semantically validated and its application evidenced the relevance, since it is considered the necessary and feasible instrument. The items with greater filling were related to the patient's characteristic being the identification the item more filled and the recommendation item had less fulfillment, because as nursing techn icians consider that some items of this variable are private to the nurse.

Keywords: Management of nursing practice; Validation studies; Hospital communication system; Nursing team.

LISTA DE TABELAS

Tabela 1 - Caracterização dos Juízes quanto ao sexo, idade, tempo de profissão, atuação profissional e formação. Botucatu, 2019.

Tabela 2 - Percentual de adequação das propriedades de cada item: clareza, pertinência e aparência. Botucatu, 2019.

Tabela 3 - Distribuição das sugestões por item avaliado. Botucatu, 2019.

Tabela 4 - Características dos participantes na aplicação do instrumento de passagem de plantão (SBAR) (n=11). Botucatu, 2019.

Tabela 5 - Dados referentes a passagem de plantão e uso da ferramenta SBAR. Botucatu, 2019.

Tabela 6 - Análise do uso da ferramenta SBAR no período de um mês (n=110). Botucatu, 2019.

Tabela 7 - Dados das variáveis preenchidas no instrumento de avaliação da passagem de plantão pelos participantes. Botucatu, 2019.

LISTA DE QUADROS

Quadro 1 – Versão 1 do instrumento construído.

Quadro 2- Descrição das sugestões efetuadas pelos Juízes. Botucatu, 2019.

Quadro 3 – Segunda versão do instrumento para aplicação na Unidade cenário da pesquisa.

Quadro 4 - Itens por ordem de preenchimento.

SUMÁRIO

1. INTRODUÇÃO...18

2. REVISÃO DA LITERATURA...19

2.1 Passagem de Plantão...19

2.2 Segurança do Paciente e a Comunicação na Passagem de Plantão..............21

2.3 Metodologia *SBAR* (*Situation, Background, Assessment, Recommendation*)..24

3. JUSTIFICATIVA DO ESTUDO......... ………………………………………...26

4- OBJETIVO……………………………………………………………………27

4.1-Objetivo Geral 27

4.2. Objetivos Específicos...27

5 – MÉTODO..28

5.1 - Tipo do Estudo...28

5.2 - Local do Estudo...28

5.3 – Participantes...29

5.4 - Procedimento de construção, validação do instrumento de coleta de dados..30

5.5 – Procedimentos Éticos..32

6. RESULTADOS...33

6.1- Construção do Instrumento..33

6.2 - Validação do instrumento...34

6.3- Avaliação dos Participantes...38

6.4- Análise do preenchimento do instrumento..41

7 - DISCUSSÃO..45

8 - CONCLUSÃO..51

REFERÊNCIAS..52

APÊNDICES………………………………………………………………………57

ANEXOS……………………………………………………………………………62

APRESENTAÇÃO

Há 12 anos atuo como Enfermeira e atualmente presto serviços a uma Instituição onde a pesquisa vista como melhoria no desenvolvimento pessoal e profissional, me motivou ainda mais em iniciar o estudo. Ao passo que adquiri experiência, percebi a necessidade de uma comunicação mais consistente e compreensiva entre os membros da equipe de enfermagem, passei por alguns momentos constrangedores por falta de informações relacionadas ao paciente, me preocupei com o prejuízo na continuidade da assistência devido a uma comunicação ineficiente, sendo essa a maior razão para iniciar a pesquisa.

Nessa perspectiva, notei urgência em melhorar a comunicação da equipe de enfermagem durante a passagem de plantão, adotando uma comunicação bem estruturada, clara e objetiva, pois acredito que facilitaria o funcionamento das rotinas assistenciais e administrativas. Durante minha atuação como pesquisadora, percebi a satisfação dos participantes, pois num determinado momento da pesquisa, foi necessário a leitura do prontuário do paciente, então o participante passou a conhece-lo como um todo e consequentemente desenvolver maior interesse no cuidado.

Construir e validar uma ferramenta de passagem de plantão, usando a metodologia SBAR, será um divisor de águas para a equipe de saúde na unidade explorada, tendo em vista o maior interesse no histórico de saúde do paciente assistido, a possível melhora do cuidado e a facilidade na comunicação durante a passagem de plantão.

Acredito que o resultado dessa pesquisa será fundamental para uma assistência de enfermagem com qualidade no hospital cenário do estudo.

1. INTRODUÇÃO

A enfermagem tem como premissa essencial o cuidado, e para realizá-lo com qualidade faz-se necessário utilizar instrumentos em seu processo de trabalho que possibilitem a articulação da teoria com a prática.

Dentre as atividades fundamentais para o trabalho na Enfermagem, a passagem de plantão é um dos elementos cruciais para a garantia da continuidade das ações que são inerentes a esse trabalho.

Dessa forma, a comunicação torna-se indispensável para que as equipes consigam planejar e executar bem suas atividades, adotando um fluxo adequado de comunicação com canais bem estruturados, facilitando o funcionamento das rotinas assistenciais e administrativas.

É por meio da comunicação que é possível partilhar mensagens recebidas e emitidas, exercendo grande influência no comportamento das pessoas de forma mútua.

O SBAR *(Situation-Background-Assessment-Recommendation)* é uma ferramenta de comunicação que consiste no preenchimento de quatro critérios: **S**: Situação, **B**: Breve Histórico, **A**: Avaliação e **R**: Recomendação, permitindo a organização da informação que está sendo emitida, tornando-a clara e padronizada, de forma que as informações essenciais do cuidado com paciente não se percam durante a troca de plantão.[1]

O uso da ferramenta SBAR possibilita o maior foco no paciente, diálogo entre a equipe mais efetivo e menor transcrição de dados. Assim, a ferramenta demonstrou ser concisa, consistente, compreensiva e com o relato focado no paciente.[2]

Embora com utilização consolidada por enfermeiros de países da América do Norte e Europa, pouca literatura existe no Brasil sobre o uso dessa ferramenta, em especial para a passagem de plantão dos profissionais de enfermagem.

2. REVISÃO DA LITERATURA

2.1 Passagem de Plantão

A passagem de plantão é um processo que faz referências à informações específicas de pacientes que são transmitidas de um profissional de saúde para outro, ou de uma equipe assistencial para outra ou ainda de profissionais de saúde para pacientes e familiares quando estes vão para a casa.[3]

Esse processo pode ocorrer em muitos cenários na assistência continuada ao paciente, incluindo: admissão a partir da assistência primária, de um médico para outro, mudança de turno da enfermagem em unidades assistenciais, relatório de enfermagem na transferência de pacientes (entre unidades ou organizações de saúde), relatório de anestesiologistas de sala operatória para salas de recuperação anestésica, comunicação em unidades de urgência e emergência com atendimento pré-hospitalar e transferência para unidades de internação e alta do paciente para casa ou outra unidade de saúde.[3]

A Organização Mundial da Saúde (OMS) elaborou estratégias que devem ser consideradas na passagem de plantão com ênfase para quatro aspectos: 1) assegurar que a organização de saúde implemente, de forma padronizada, a comunicação na passagem de plantão sugerindo o uso da técnica SBAR para essa comunicação. Considerar a alocação de tempo suficiente para que as informações importantes sejam comunicadas e a não interrupção durante esse processo. Informações referentes a condição do paciente, medicamentos, planos de tratamento e mudanças na condição do paciente são essenciais; 2) assegurar que organizações de saúde implementem sistemas que garantam a alta do paciente com todas as informações necessárias ao seu tratamento, como por exemplo, diagnóstico, plano de tratamento, medicamentos e resultados de exames; 3) Incorporar treinamento na comunicação da passagem de plantão de forma permanente; 4) encorajar a comunicação entre a organização de saúde e os prestadores de cuidado (formal e informal).[3]

Na Enfermagem a passagem de plantão é uma prática essencial, pois refere-se a informações fundamentais na troca de turno com a finalidade de transmitir essas informações entre a equipe de enfermagem de forma objetiva e concisa, abordando os acontecimentos referentes à assistência direta e/ou indireta ao paciente, durante o turno de trabalho, assim como assuntos de interesse institucional.[4]

Considerada fundamental no processo de trabalho da enfermagem, visto que neste momento ocorre troca de informações entre a equipe que prestou assistência com a equipe que assumirá os cuidados no turno seguinte. É por meio da passagem de plantão que o enfermeiro terá uma visão geral sobre o cenário que assumirá, além de diagnosticar, planejar, delegar e aplicar as intervenções necessárias.[5 6]

O sucesso da passagem de plantão depende de um trabalho de equipe bem articulado, criando formas alternativas e eficazes para a transmissão de informações consistentes e de qualidade que dependerá habilidade de quem fala, da modalidade escolhida, do tempo despendido e da preocupação da equipe em registrar informações que relatem as intercorrências com o paciente. Essa ação visa um momento de reflexão e entrosamento entre a equipe, sob o enfoque multidisciplinar, reavaliação das condutas, proporcionando crescimento e desenvolvimento mútuo.[4]

Cada instituição e cada profissional têm sua dinâmica para realizar a passagem de plantão. O comprometimento e a valorização dessa ação devem ficar claras ao profissional, para que a transmissão da informação seja produtiva e eficaz, independentemente do método que se utiliza ou do tipo de unidade de prestação de serviços.[5 6 7]

Portanto, é um recurso estratégico para a coordenação do cuidado de enfermagem com possibilidade de resultados positivos a continuidade da assistência.[8]

No entanto, fatores como quantidade excessiva ou reduzida de informações, pouca possibilidade de realizar questionamentos durante o processo, informações inconsistentes, omissões ou informações erradas, não existência de processos padronizados, registros falhos e ilegíveis, interrupções e distrações, além de uma equipe não preparada para esse processo, são aspectos que dificultam essa prática com prejuízo para o paciente.[9]

O Código de Ética dos Profissionais de Enfermagem em seu Artigo 38 da Sessão II das relações com os trabalhadores de enfermagem, saúde e outros, dispõe entre as responsabilidades e deveres desses profissionais:

> **Art. 38** Prestar informações escritas e/ou verbais, completas e fidedignas, necessárias à continuidade da assistência e segurança do paciente.[10]

Explicita–se assim, a legalidade desse processo para a Enfermagem, visto sua importância no contexto da saúde.

2.2 Segurança do Paciente e a Comunicação na Passagem de Plantão

A segurança do paciente tem como objetivo reduzir os riscos assistenciais e atualmente encontra-se em pauta no cenário mundial. Desta forma, tornou-se característica fundamental para uma assistência de qualidade e uma questão estratégica no contexto do cuidado.

O avanço e fortalecimento da cultura de segurança do paciente são percebidos nas instituições hospitalares seja por meio da implantação de programas de qualidade, de indicadores e protocolos assistenciais além da disseminação das acreditações hospitalares. A prática segura ocupou um espaço que era ocupado culturalmente punitiva e o medo de errar, gerando benefícios para o paciente e para a equipe que presta cuidados baseados em evidências e com o respaldo das melhorias de processo. As práticas clínicas pautadas por processos bem desenhados com sistemas e ferramentas que previnem a ocorrência de eventos adversos com a adoção de medidas de barreiras preventivas. O erro humano deixou de ser tratado focando no indivíduo e passa a ser tratado com foco no processo, “despersonificando” as falhas processuais.[11]

Em 1999, houve a publicação do livro intitulado *To err is human: building a safer health care system,* pelo *Institute of Medicine*, nos Estados Unidos da América, mostrando uma preocupação inicial com a segurança do paciente, evidenciando as necessidades de mudanças. Este livro apontou os dados de mortalidade decorrentes de erros evitáveis no cuidado à saúde e os seus custos.[11 12]

Desde a 55ª Assembleia Mundial da Saúde realizada em 2002, o tema tornou-se prioridade para a OMS). Com a finalidade de facilitar o desenvolvimento de práticas e políticas de pacientes em diversos países, por meio de programas, criou-se em 2004 a Aliança Mundial para a Segurança do Paciente, fato que ocorreu na 57ª Assembleia Mundial da Saúde.[13]

As metas internacionais dessa Aliança, visando a qualidade e segurança do paciente. Dentre elas, podemos citar aquela referente à comunicação efetiva que reforça a necessidade de melhorar o processo de transmissão de informações entre os profissionais da saúde, limita a ocorrência de erros, beneficiando a segurança do paciente.[1415]

A quebra na continuidade do tratamento e na qualidade da assistência decorrente da má comunicação, evidencia prejuízos relevantes, abrindo uma lacuna entre a qualidade e a assistência.[16]

O propósito da enfermagem é proporcionar o cuidado, com objetivo de manter a integridade humana, em forma de rede de cooperação e de participação coletiva no processo de gestão e, quando compreendermos a relevância do valor dos sujeitos envolvidos, o atendimento será alinhado à Política Nacional de Humanização.[17]

Portanto, caracteriza-se como elemento fundamental, a comunicação entre os profissionais, os gestores e os clientes, assim, a transversalidade é uma diretriz estabelecida desta política.[17]

Desta forma, a comunicação torna-se um componente imprescindível para o cuidado e como alicerce das relações interpessoais, em suas variadas formas, tem um papel de instrumento de significância humanizadora e, para tal, a equipe precisa estar determinada e comprometida para consolidar essa relação e entender que é fundamental reconhecer o cliente como sujeito do cuidado e não passivo a ele.

O diálogo é um método importante e destaca-se pela melhoria que traz ao realizar o cuidado de enfermagem, pois além de criar afinidade entre as pessoas, estabelece uma proximidade integrada de culturas, e troca de experiências e vivências.[18]

Uma comunicação efetiva entre os membros da equipe de enfermagem e multidisciplinar favorece as inter-relações profissionais estabelecidas no trabalho e possibilitam delimitar melhor se a assistência ao paciente será ou não humanizada. Para a percepção contínua da realidade do paciente e do profissional é imprescindível estreitar os laços de comunicação, para o processo de humanização.[19]

Assim sendo, é possível identificar e perceber os problemas decorrentes da comunicação, colaborando com a interação profissional e pessoal, uma vez que, o homem se utiliza da comunicação em todos os momentos e ações de sua vida, e é por meio dela que se dividem as experiências e pode-se modificar a si mesmo e o contexto em que está entreposto.[20] [21] Melhorar a comunicação entre os membros da equipe de enfermagem é entender os métodos de um processo, isso contribuirá não somente para o desempenho significativo para com o usuário, mas também estabelecerá um bom relacionamento entre os membros da equipe.[22]

À luz desse pensamento, identificar o processo de comunicação a partir de uma definição complexa, nas suas variadas formas e por ser um fenômeno que possui componentes específicos, de modo que busque melhorar a compreensão e a interação entre os partícipes da comunicação, se faz necessário para poder, de algum modo, contribuir com o relacionamento entre os indivíduos. Reforça-se a importância desse relacionamento entre trabalhadores que possuem o mesmo objetivo, quando se fala em assistência de enfermagem, ou seja, do cuidado.

No entanto, as dificuldades de comunicação entre os profissionais de saúde podem causar danos e aumentar os riscos de incidentes durante o tratamento oudurante a passagem de plantão e por isso necessitam ser analisadas de forma contínua.[23]

Torna-se muito importante o desenvolvimento de competência em comunicação interpessoal, pois é uma habilidade a ser adquirida pelo enfermeiro que proporcionará um cuidar consciente, verdadeiro e transformador.[24]

É fundamental que questões que relacionam a segurança do paciente e a comunicação sejam discutidas nas organizações de saúde, em especial no que se refere à passagem de plantão, pois por esse processo de trabalho da enfermagem, fortalece a comunicação da equipe onde são repassadas relevantes informações com a finalidade de manter a continuidade do cuidado e a garantia da segurança do paciente.[9 25]

Diante desses apontamentos, a passagem de plantão de enfermagem pode ser considerada uma atividade essencial para a comunicação, auxiliando na continuidade da assistência.[26] Por sua vez, esta prática visa transmitir informações de forma objetiva, clara e concisa sobre os acontecimentos ocorridos durante um determinado turno de trabalho, permitindo aos profissionais ter uma visão geral do setor, assim da evolução dos pacientes, o que facilitará o planejamento e a organização de atividades.[27]

Assim, a passagem de plantão pressupõe que a comunicação seja efetiva e eficaz, pois trata-se de uma competência indispensável para que a assistência seja continuada pela equipe de enfermagem com vistas a preservação e restauração da saúde.[8]

2.3 Metodologia *SBAR* (*Situation, Background, Assessment, Recommendation*)

A técnica *SBAR* (*Situation-Background-Assessment-Recommendation*) – desenvolvida no *Kaiser Permanent of Colorado* (Evergreen, CO) – proporciona uma estrutura para a comunicação entre os membros da equipe de atendimento de saúde.

O *Institute for Health Improvement* [28], descreve o *SBAR* como um método efetivo, descomplicado e fácil de memorizar, que pode ser usado para adequar conversas, porém, exige atenção e ação imediata, tornando possível também o trabalho em equipe. Expectativas são estipuladas para o quê e como será comunicado entre os membros da equipe, de modo compreensivo e aplicado, favorecendo a cultura de segurança do paciente.

A ferramenta compreende questionamentos rápidos e padronizados, avaliando 4 critérios, para que todos compartilhem de informações precisas e focadas, reduzindo a necessidade de repetição, sendo possível elaborar informações detalhadas.[29]

Essa metodologia que estrutura a comunicação entre a equipe de enfermagem de forma organizada, clara e objetiva, preenche 4 critérios representados por:

S – *situation* - situação

B – *background* - breve histórico

A – *assessment* - avaliação

R – *recommendation* - recomendação

Na “Situação - *situation*”, estrutura-se um relato conciso da situação do paciente.

Em “Breve Histórico - *background*”, relatam-se as informações pertinentes do caso do paciente como história prévia, hipótese diagnóstica, entre outros.

Na “Avaliação - *assessment*” estratifica-se o caso clínico do paciente, fornecendo dados reais que suportem as tomadas de decisão.

E, em “Recomendação - *recommendation*- ”, o enfermeiro recomenda ações à equipe de enfermagem mediante a análise das necessidades do paciente.[1]

A utilização dessa técnica ou ferramenta pode contribuir para que os erros de comunicação diminuam e fatores contribuintes da melhoria de atitudes de segurança sejam incrementados, pois é uma forma padronizada de passagem de plantão que é válida para a comunicação entre os membros da equipe de saúde.[30]

As técnicas de comunicação estruturadas como o SBAR melhoram a percepção e a colaboração necessária entre os membros da equipe de saúde, no processo de passagem de plantão.[31]

Na enfermagem, a utilização da ferramenta SBAR é mais recente, especialmente fora do Brasil, e tem evidenciado tratar-se de uma comunicação efetiva, pois dá segurança para a equipe de enfermagem pela acurácia das informações, além de ser simples e contribuir para a segurança do paciente.[32]

Estudos conduzidos nos Estados Unidos demonstrou que o uso da técnica SBAR para passagem de plantão da equipe de enfermagem promoveu melhor estrutura, consistência, priorização, acurácia e compreensão das informações necessárias ao cuidado. Além disso, o uso da técnica possibilitou melhor comunicação e conhecimento sobre os pacientes assistidos.[1 2]

3. JUSTIFICATIVA DO ESTUDO

A justificativa para a realização desse estudo, se pauta no fato de que, no momento não há uma passagem de plantão padronizada para a equipe de enfermagem no cenário a ser estudado. Dessa forma, este estudo tem como finalidade construir um instrumento de passagem de plantão com base no SBAR recomendada pela OMS, para melhorar a qualidade da informação transmitida entre os turnos e até mesmo entre os setores, como por exemplo, na transferência de pacientes, resultando na continuidade da assistência.

Considerando o *SBAR* uma ferramenta que possibilita uma melhor comunicação entre profissionais de saúde e a escassez de estudos no Brasil que utilizam essa ferramenta para a passagem de plantão, questiona-se: A construção de um instrumento de passagem de plantão poderá contribuir para melhorar a qualidade da comunicação, referentes aos pacientes na passagem de plantão?

A construção e a validação de um instrumento que melhore a comunicação entre as equipes de enfermagem, é, portanto, a maior contribuição deste estudo.

4- OBJETIVO

4.1-Objetivo Geral

Construir e realizar a validação semântica de um instrumento de passagem de plantão utilizando a metodologia *SBAR.*

4.2. Objetivos Específicos

- Realizar revisão da literatura sobre ferramenta *SBAR*;
- Constituir o instrumento para a passagem de plantão;
- Realizar a validação semântica do instrumento construído;
- Realizar a aplicação do instrumento após a validação semântica.

5 - MÉTODO

5.1 - Tipo do Estudo

Trata-se de estudo metodológico cujo objetivo é trabalhar com instrumentos e ferramentas complexas e desenvolvimento de referenciais metodológicos.[33]

A proposta foi construir e validar semanticamente um instrumento para passagem de plantão padronizada. A pesquisa metodológica aborda o desenvolvimento e validação do instrumento em uma determinada área de assistência de enfermagem. Esse tipo de estudo abrange quatro etapas, a saber: definição do conceito ou comportamento a ser medido, formulação dos itens do instrumento, desenvolvimento de instruções para o teste de confiabilidade e da validade do instrumento de medida.[34]

5.2 - Local do Estudo

O estudo foi realizado com juízes em dois cenários, ensino e assistência na enfermeira de Gastroenterologia cirúrgica em um Hospital público de ensino - Hospital das Clínicas – Faculdade de Medicina de Botucatu.

Trata-se de um hospital geral que realiza atividades de ensino, pesquisa e extensão. É referência no Sistema Único de Saúde em âmbito municipal, estadual e nacional, com atendimento à pacientes que demandam assistência de alta complexidade.[35]

O setor onde foi realizada a pesquisa assiste pacientes submetidos às diversas cirurgias do aparelho digestório, a enfermaria possui 28 leitos e equipe multidisciplinar composta por: enfermeiros, técnicos de enfermagem, auxiliares de enfermagem, médicos especialistas e médicos residentes, psicólogos, assistente social, nutricionistas e fisioterapeutas.

O quadro de funcionários atual da equipe de enfermagem consiste em sete enfermeiros, 18 técnicos de enfermagem e três auxiliares de enfermagem.

O processo de passagem de plantão entre os membros da equipe de enfermagem atual acontece da seguinte forma:

O momento da passagem de plantão, envolve basicamente duas equipes: a que está encerrando o turno de 12 horas e a que está iniciando um novo, pelo mesmo período.

A passagem de plantão acontece em forma de reunião, algumas informações consideradas relevantes pela equipe, são anotadas em um papel "rascunho", cuja inutilização se dá logo após a troca do plantão, por vezes alguns profissionais optam apenas por uma passagem de plantão de forma oral, importante ressaltar que os técnicos de enfermagem ficam responsáveis por pacientes conforme escala diária, sendo que cada técnico fica escalado com média de oito a nove pacientes por plantão.

Entretanto, observa-se que não há o envolvimento efetivo das duas equipes, os profissionais que encerram e que iniciam a jornada nem sempre estão presentes na passagem de plantão, ficando diversas vezes apenas dois profissionais de cada turno, responsáveis pela transmissão das informações. Constantemente, a atividade da passagem de plantão é interrompida para que as informações sejam repetidas, devido interferências ou falta de atenção dos envolvidos. Impontualidade, conversas paralelas, relatos de informações não relevantes, falta de atenção e falhas na comunicação, tornam o tempo dessa atividade indevido, tornando–se cansativo e maçante, gerando perda de informações importantes à assistência, como, por exemplo, cuidados eventualmente esquecidos ou não realizados conforme o planejado.

5.3 – Participantes

Participaram da validação semântica do instrumento construído 10 Juízes, enfermeiros especialistas com experiência na atuação clínica.

Para aplicação do instrumento após sua validação por juízes, 11 técnicos de enfermagem do quadro funcional da enfermaria de Gastroenterologia cirúrgica do hospital participaram dessa etapa da pesquisa. Os enfermeiros não foram incluídos nessa etapa, pois participaram da validação do instrumento.

Os critérios de inclusão dos participantes forami: ser técnico de enfermagem, ambos os sexos, com qualquer idade, independentemente do tempo de atuação, que prestasse cuidados diretos aos pacientes e que participasse da passagem de plantão.

Do grupo de técnicos de enfermagem, foram excluídos do estudo 10 profissionais: cinco profissionais não exercem atividades de cuidado direto aos pacientes, portanto, não realizam a passagem de plantão, três estavam de férias, um estava de licença saúde no momento da pesquisa e um não aceitou participar do estudo, alegando não ter tempo.

5.4 -Procedimento de Construção, Validação do Instrumento e Coleta dos Dados

Foi construído um instrumento de passagem de plantão considerando a ferramenta SBAR por meio do levantamento da literatura e das características da enfermaria de Gastroenterologia cirúrgica do hospital.

Após a construção e organização do instrumento, tem-se uma hipótese sobre a possibilidade de representar adequadamente o referido instrumento. Sendo uma hipótese, é necessário testá-la em relação à avaliação de outras pessoas, que possam assegurar sua validação. Para realizar esse tipo de avaliação há a possibilidade de avaliação dos juízes e análise semântica.[36]

Em março de 2019 foi encaminhado aos juízes, enfermeiros especializados em assistência, o instrumento construído para a validação semântica considerando o Índice de Validade de Conteúdo (IVC) maior ou igual a 80%. Os juízes foram divididos em dois grupos: o primeiro grupo foi composto por seis enfermeiros com experiência profissional na prática clínica, o segundo grupo representado por quatro docentes de enfermagem com atuação em ensino, pesquisa e extensão na área clínica.

O objetivo dessa avaliação segundo a concepção de Pasquali (2010) é verificar a adequação da representação do atributo, pois visa identificar se o instrumento é acessível e facilmente compreendido por profissionais com diferentes níveis de conhecimentos e habilidades.

Embora obtendo um Índice de Validade de Conteúdo (IVC) de 91,7%, as sugestões foram analisadas pela pesquisadora principal e orientadora e as pertinentes foram incorporadas ao instrumento.

A aplicação do instrumento deu-se no período de um mês (fevereiro de 2019) na enfermaria da Gastroenterologia cirúrgica do hospital cenário do estudo.

Cada participante recebeu um caderno simulado, contendo o modelo proposto, foram instruídos a preencher durante o turno e um modelo ficou disponível para cada profissional individualmente por 28 dias.

Em seguida, houve a aplicação do instrumento na passagem de plantão na enfermaria referida, e posteriormente os participantes avaliaram o instrumento construído, respondendo questionário elaborado pela pesquisadora e orientadora.

5.5 – Procedimentos Éticos

O projeto desta investigação foi submetido ao Comitê de Ética em Pesquisa da Faculdade de Medicina de Botucatu da Universidade Estadual Paulista "Júlio de Mesquita Filho" e aprovado sob o número do parecer 3.009.068 e CAAE 02168918.0.0000.5411 (Anexo 1).

Os Juízes e equipe de enfermagem foram convidados a participar do estudo, garantindo o anonimato e o esclarecimento de todas as etapas da pesquisa. Os que aceitaram participar assinaram o Termo de Consentimento Livre e Esclarecido (TCLE) de acordo com a resolução 466/12 do Conselho Nacional de Saúde[1] (Apêndice 1).

---

[1] Ministério da Saúde. Conselho Nacional de Saúde. Resolução nº 466, de 12 de dezembro de 2012. Aprova as seguintes diretrizes e normas regulamentadoras de pesquisas envolvendo seres humanos.

6- RESULTADOS

6.1- Construção do Instrumento

O instrumento foi construído com base na literatura sobre o SBAR e na especificidade da Unidade de Internação cenário do estudo.

Os itens constantes do instrumento foram: dados de identificação do paciente; Riscos identificados; Situação (S); Breve história (B); Avaliação (A); Recomendação (R).

Esses itens visaram abranger elementos essenciais para a passagem de plantão possibilitando a continuidade da assistência de enfermagem.

A versão 1 do instrumento construído é apresentado no quadro 1.

VERSÃO 1 - INSTRUMENTO DE PASSAGEM DE PLANTÃO - SBAR

UNIDADE DE INTERNAÇÃO:____
DATA: Data de Admissão:
LEITO: DN/Idade: Sexo: M ( ) F ( )
NOME DO PACIENTE:____

<table>
<tr><th>INDICADORES</th><th>SITUAÇÃO
(S)</th><th>BREVE HISTÓRIA
(B)</th><th colspan="3">AVALIAÇÃO
(A)</th><th>RECOMENDAÇÃO
(R)</th></tr>
<tr><td rowspan="3">Queda ( ) Sim ( ) Não
TEV ( ) Sim ( ) Não
Lesão pressão ( ) Sim ( ) Não
Outros:

SCP:
Identificação:
Pulseira ( ) Sim ( ) Não
Placa leito ( ) Sim ( ) Não
Acompanhante ( ) Sim ( ) Não</td><td rowspan="3">DI:
Diagnóstico Médico: ______________
____
____
Médico Responsável:

Diagnósticos de Enfermagem/Levanta-Mento de problemas de Enfermagem:
Nas últimas 24 horas
____
____
____
____
____
____
____
____
____
____
____
____
____
____
____
____
Enfermeiro Responsável:</td><td rowspan="3">Alergias: ______________
____
____

Co-morbidades:
____
____
____
____
Histórico cirúrgico:
____
____
____

Isolamento/Precauções:
____
____
____

Barreiras para comunicação:
____
______________</td><td>Sinais Vitais:
T=
FC=
FR=
PA=
Oxigenação/
Ventilação
____
______________</td><td>Ex. Laboratoriais
____
____
____
____

Nutricional:
____
____
____
______________</td><td>Mucosas
____
____

Consciência
____
____

Locomoção
____
______________</td><td rowspan="2">Interconsultas:
____
____
____
____

Intervenções Enfermagem:
____
____
____
____
____
____
____
____

Outros
____
____
____
____
____
____
____
______________</td></tr>
<tr><td>AVP

CVC

Drenos
____
____
SNG:
SNE:</td><td>Curativos/Incisão
____
____

Estomia
____
____

NPP:</td><td>Eliminações:
____
____

HGT:

Escalas:
____
____
____
______________</td></tr>
<tr><td>Medicamentos
IV: ______________
IM:______________
______________</td><td colspan="2">Intercorrências:
____
____
____
______________</td><td>Assinatura:

______________</td></tr>
</table>

6.2- Validação do Instrumento

O instrumento foi encaminhado aos Juízes para validação por meio de um impresso que analisou a clareza, ou seja, atributo daquilo que é inteligível, fácil de ser compreendido ou entendido; pertinência (característica do que é concernente, apropriado e relevante) e aparência (aspecto ou aquilo que se mostra superficialmente ou à primeira vista). (Apêndice 2).

Os juízes foram predominantemente do sexo feminino (80%), com atuação profissional em docência (40%) e possuíam pós-graduação (60%). A caracterização dos Juízes está apresentada na tabela 1.

Tabela 1- Caracterização dos Juízes quanto ao sexo, idade, tempo de profissão, atuação profissional e formação. Botucatu, 2019.

VARIÁVEL	N	%
Sexo		
Sexo feminino	08	80,0
Sexo masculino	02	20,0
Idade	Média 42,5	DP ± 12.0
Tempo de Profissão	Média 16,1	DP ± 12,4
Atuação Profissional		
Gestão	03	30,0
Assistência	03	30,0
Docência	04	40,0
Formação		
Graduação	04	40,0
Especialização	02	20,0
Mestrado	01	10,0
Doutorado	03	30,0
Pós-Doutorado	01	10,0

O Índice de Validade de Conteúdo (IVC) foi igual a 91,7%. Destacando que os itens: clareza na identificação, situação e histórico; pertinência nos indicadores; aparência na situação e histórico, tiveram 100% de concordância. A tabela 2 apresenta todos os itens avaliados e sua concordância.

Tabela 2- Percentual de adequação das propriedades de cada item: clareza, pertinência e aparência. Botucatu, 2019.

PROPRIEDADE / ÌTEM	%
Clareza na identificação	100
Pertinência na identificação	90
Aparência na identificação	80
Clareza nos indicadores	80
Pertinência nos indicadores	100
Aparência nos indicadores	90
Clareza sobre a situação	100
Pertinência sobre a situação	90
Aparência sobre a situação	100
Clareza do histórico	100
Pertinência do histórico	90
Aparência do histórico	100
Clareza na avaliação	80
Pertinência na avaliação	90
Aparência na avaliação	80
Clareza nas recomendações	90
Pertinência nas recomendações	90
Aparência nas recomendações	90
Média	91,1

Os Juízes também realizaram sugestões para adequação dos itens propostos no instrumento. As sugestões mais frequentes (26,8%) referiram-se ao item avaliação. Essas sugestões estão descritas na tabela 3.

Tabela 3. Distribuição das sugestões por item avaliado. Botucatu, 2019.

ITEM MENCIONADO EM SUGESTÕES DE MELHORA	N	%
**Avaliação**	11	26,8
**Histórico**	7	17,0
**Identificação**	6	14,6
**Indicadores**	6	14,6
**Recomendações**	6	14,6
**Situação**	5	12,2
Total	**41**	**100**

Para melhor compreensão das sugestões efetuadas e quais delas foram acatadas, o quadro 2 as apresenta de forma detalhada assinalando-as com a letra “x”.

Quadro 2- Descrição das sugestões efetuadas pelos Juízes. Botucatu, 2019.

DESCRIÇÃO DAS SUGESTÕES	ACATADAS
Incluir número do Registro	X
Demonstrar mais clareza nos itens	X
Substituir “Recomendação” por Recomendações	
Incluir dias de internação	X
Colocar os itens sobrepostos com alternativas sim e não	X
Substituir “Indicadores” por Riscos Identificados	X
Substituir “Situação” por Diagnóstico ou Condições de Saúde	
Incluir diagnóstico referido pelo paciente	
Incluir procedência do paciente	X
Incluir fármacos de controle especial	X
Inverter as vias e administração das medicações	X
Inserir escala de dor	X
Incluir dia do Pós-Operatório	X
Incluir datas dos drenos	X
Incluir datas das trocas das bolsa e sondas	X
Incluir especialidade médica responsável pelo paciente	X
Incluir grau de dependência para o cuidado	X
Incluir campo com alternativas Pré e Pós-Operatório	X
Excluir exames laboratoriais	X
Substituir “ Interconsulta” por Interconsulta de Enfermagem	X
Incluir escala de Braden	
Incluir escala de Qualidade da Assistência	X
Incluir antecedentes pessoais	
Excluir Interconsulta	
Colocar itens em *Check List*	X
Maior clareza nas abreviações	X
Manter Diagnósticos de Enfermagem mais usados	X
Incluir Diagnóstico Médico atual	X
Minimizar poluição visual do impresso	X
Melhorar formatação	X
Acrescentar religião	X
Acrescentar fármacos mais usados	X
Mudar a ordem dos itens	X
Incluir vínculo familiar, visitas recebidas	X
Inserir campo para assinatura do profissional.	

Após análise e sugestões dos Juízes, embora o IVC tenha sido 91,7%, considerou-se realizar as alterações julgadas pertinentes no instrumento que não influenciaram no método. Assim, o instrumento para aplicação na Unidade cenário da pesquisa está descrito no quadro 3.

INSTRUMENTO DE PASSAGEM DE PLANTÃO - SBAR
UNIDADE DE INTERNAÇÃO: ENFERMARIA DE GASTROENTEROLOGIA CIRÚRGICA

NOME DO PACIENTE:____ LEITO:____ RG:____ DN:____ IDADE:____

DATA:____ DATA DE ADMISSÃO:____ DI:____ SEXO: M ( ) F ( ) RELIGIÃO:____

RISCOS IDENTIFICADOS

HIGIENE/ CONFORTO
( ) BANHO LEITO
( ) BANHO CADEIRA
( ) BANHO ASPERSÃO
AUXILIADO ( ) SIM
( ) NÃO

IDENTIFICAÇÃO
PULSEIRA
( ) SIM ( ) NÃO

PLACA LEITO
( ) SIM ( ) NÃO

TEV
( ) SIM ( ) NÃO

FUGULIN (SCP)
( ) SIM ( ) NÃO

QUALIDADE ASSIST.
( ) SIM ( ) NÃO

RISCO QUEDA
( ) SIM ( ) NÃO

LESÃO PRESSÃO
( ) SIM ( ) NÃO

ACOMPANHANTE
( ) SIM ( ) NÃO

VISITA FAMILIAR
( ) SIM ( ) NÃO

SITUAÇÃO (S)

PRÉ OP. ( ) PÓS OP. ( ) CLÍNICO ( )

DIAGNÓSTICO MÉDICO ATUAL
____

MÉDICO RESPONSÁVEL
____

ESPECIALIDADE
____

DIAGNÓSTICOS DE ENFERMAGEM/LEVANTA-MENTO DE PROBLEMAS DE ENFERMAGEM NAS ÚLTIMAS 24 HORAS
____

BREVE HISTÓRIA (B)

ALERGIAS
____

CO-MORBIDADES
____

MEDICAÇÕES DE USO CONTÍNUO
____

HISTÓRICO CIRÚRGICO
____

ISOLAMENTO/PRECAUÇÕES
____

BARREIRAS PARA COMUNICAÇÃO
( ) SIM ( ) NÃO
QUAIS____

AVALIAÇÃO (A)

MUCOSA
____

CONSCIÊNCIA
____

LOCOMOÇÃO
____

OXIGENAÇÃO/ VENTILAÇÃO
____

NUTRICIONAL
____
SNG:____
____
DATA____

SNE:____
____
DATA____

GTT:____
____
DATA____

VO ( ) NPP ( )

SSVV
T:____
FC:____
FR:____
PA:____

DOR:____

HGT:____

REPOSIÇÃO
____

AVP____

DATA____

CVC____

DRENOS
____

CURATIVOS INCISÃO
____

ESTOMIA
____

Eliminações:
____

Escalas:
____

MEDICAMENTOS
IV ( )
____
VO ( )
____
IM ( )
____

MEDICAMENTOS
ATB____

DVA____

OUTROS____

RECOMENDAÇÃO (R)

INTERCONSULTAS DE ENFERMAGEM
____

INTERVENÇÕES ENFERMAGEM
____

OUTROS/INTERCORRÊNCIAS
____

ENFERMEIRO (SAINDO)
____

ENFERMEIRO (CHEGANDO)
____

6.3- Avaliação dos Participantes

Os participantes aplicaram 351 instrumentos de passagem de plantão no mês de fevereiro (do dia primeiro ao 28º dia) e após a aplicação responderam um questionário elaborado pela pesquisadora e orientadora com a finalidade de avaliar a passagem de plantão e o uso da ferramenta SBAR (Apêndice 3).

A caracterização dos participantes demostrou serem predominantemente do sexo feminino (90,9%) e com formação em nível técnico (81,8%). A tabela 4 descreve essa caracterização.

Tabela 4- Características dos participantes na aplicação do instrumento de passagem de plantão (SBAR) (n=11). Botucatu, 2019.

VARIÁVEL	N	%
Sexo		
Sexo feminino	10	90,9
Sexo masculino	01	9,1
Idade	Média 37,9 anos	
Tempo de Profissão	Média 10,3 anos	
Formação		
Nível Técnico	09	81,8
Nível Superior	02	18,2

Os participantes responderam ao questionário referente à passagem de plantão e a ferramenta SBAR. Destaca-se que a modalidade de passagem de plantão predominante foi a oral (63,6%), o local de passagem de plantão foi a sala de enfermagem (100%), o tempo estimado foi de seis a dez minutos (72,7%), o registro da passagem de plantão no livro ou prontuário e não ser esse momento para esclarecimento de dúvidas foi referido por 54,5% dos participantes, 90,9% informaram que o colega presta atenção durante a passagem de plantão, no entanto, referiram haver saídas antecipadas e atrasos que interferem nesse processo (72,7%) e que há conversas paralelas (90,9%).

Ressalta-se que 100% dos participantes consideraram que a ferramenta SBAR utilizada possui informações necessárias e 81,8% avaliaram como boa e muito boa a utilização dessa ferramenta na passagem de plantão sendo viável sua implantação para uso na unidade. Quanto as informações necessárias na passagem de plantão, as intercorrências foram referidas por 100% dos participantes.

A tabela 5 descreve os dados coletados no questionário.

Tabela 5- Dados referentes a passagem de plantão e uso da ferramenta SBAR. Botucatu, 2019.

VARIÁVEL	N	%
Modalidade de passagem de plantão		
Oral	07	63,6
Escrita	0	0,0
Oral e Escrita	04	36,4
Local que ocorre a passagem de plantão		
Ao lado do paciente	0	0,0
Corredor da Unidade	0	0,0
Sala de Enfermagem	11	100,0
Outro	0	0,0
Tempo utilizado para a passagem de plantão		
Até 5 minutos	02	18,2
6 a 10 minutos	08	72,7
11 a 20 minutos	01	9,1
21 a 30 minutos	0	0,0
Acima de 30 minutos	0	0,0
Registro das informações livro/prontuário		
Sim	06	54,5
Não	05	45,5
Momento da passagem de plantão para esclarecer dúvidas		
Sim	05	45,5
Não	06	54,5
Comportamento do colega na passagem de plantão		
Presta atenção	10	90,9
Conversas paralelas	0	0,0
Realiza procedimentos	07	63,6
Atrasos, saídas antecipadas interferindo na passagem de plantão		
Sim	08	72,7
Não	03	27,3
Interferências na passagem de plantão		
Interrupções	06	54,5
Conversas paralelas	10	90,9
Ruídos	03	27,3
Questionamentos	01	9,1
Necessidade de repetir informações	03	27,3
Outros	0	0,0
SBAR possui informações necessárias		
Sim	11	100,0
Não	0	0,0
Avaliação da passagem de plantão com SBAR		
Ruim	0	0,0
Regular	02	18,2
Boa	06	54,5
Muito boa	03	27,3
Excelente	0	0,0
Informações necessárias para a passagem de plantão		
Intercorrências	11	100,0
Condição clínica	09	81,2
Questões administrativas	0	0,0
Exames	09	81,2
Medicações	10	90,9

Alterações no tratamento	07	63,6
Identificação do paciente	07	63,6
Família e acompanhantes	04	36,4
Cuidados e procedimentos	08	72,7
Outros	0	0,0
Viabilidade da implantação do SBAR na Unidade		
Sim	09	81,8
Não	02	18,2

6.4- ANÁLISE DO PREENCHIMENTO DO INSTRUMENTO

Para a análise dos instrumentos preenchidos na passagem de plantão no período de um mês, optou-se por considerar dez instrumentos preenchidos por participante, totalizando 110 análises, sendo estas selecionadas aleatoriamente, mas contemplando o início, meio e fim do mês referido.

As variáveis analisadas no instrumento foram: 1) características dos pacientes com dez itens; 2) riscos identificados com dez itens; 3) situação com seis itens; 4) breve história com cinco itens; 5) avaliação com 15 itens; 6) Recomendação com quatro itens.

A análise considerou os itens preenchidos ou não. Quanto ao item característica do paciente, o nome foi preenchido em 100% dos instrumentos e a religião em apenas 18,2%. No item riscos identificados, acompanhantes foram preenchidos em 81,8% e não houve preenchimento na qualidade da assistência. Em situação o diagnóstico médico foi preenchido em 90,0% dos instrumentos e o diagnóstico de enfermagem não foi preenchido. No item breve história as barreiras da comunicação foram as mais preenchidas (80,0%) e o histórico cirúrgico foi o menos preenchido (15,5%). Na avaliação, as vias de administração de medicações foram preenchidas em 81,8% dos instrumentos e aparência das mucosas em 8,2%. No item recomendação, a assinatura foi preenchida por 42,7% e a intercorrência por 11,8%. A tabela 6 apresenta a análise realizada.

Tabela 6. Análise do uso da ferramenta SBAR no período de um mês (n=110). Botucatu, 2019.

**Item**	**média**	**dp**	**mediana**	**min**	**Max**
**Característica do paciente**					
Nome	100,0	0,0	100,0	100,0	100,0
Leito	94,5	12,1	100,0	60,0	100,0
RG	90,9	21,2	100,0	30,0	100,0
Data de Nascimento	83,6	32,9	100,0	0,0	100,0
Idade	85,5	25,8	100,0	30,0	100,0
Data do Dia	92,7	21,0	100,0	30,0	100,0
Data da Admissão	90,0	23,7	100,0	20,0	100,0
Dia de Internação	81,8	25,6	90,0	20,0	100,0
Sexo	90,0	26,8	100,0	10,0	100,0
Religião	18,2	26,4	10,0	0,0	90,0

**Riscos identificados**					
Higiene e Conforto	56,4	46,1	80,0	0,0	100,0
Identificação (pulseira)	80,0	34,1	100,0	0,0	100,0
Placa de Leito	80,0	36,1	100,0	0,0	100,0
Protocolo de TEV	0,9	3,0	0,0	0,0	10,0
Escala de Fugulin	0,9	3,0	0,0	0,0	10,0
Qualidade de Assistência	0,0	0,0	0,0	0,0	0,0
Risco de Queda	10,9	20,2	0,0	0,0	50,0
Lesão por Pressão	77,3	32,0	90,0	0,0	100,0
Acompanhante	81,8	29,9	100,0	10,0	100,0
Visita Familiar	80,9	29,5	90,0	10,0	100,0
**Situação**					
Pré ou Pós Operatório, Clínico	78,2	25,2	90,0	30,0	100,0
Diagnóstico Médico	90,0	20,5	100,0	40,0	100,0
Médico Responsável	42,7	41,7	50,0	0,0	100,0
Especialidade	87,3	22,8	100,0	30,0	100,0
Diagnósticos de Enfermagem	0,0	0,0	0,0	0,0	0,0
**Breve história**					
Alergias	66,4	38,5	90,0	10,0	100,0
Co-morbidades	50,0	40,5	30,0	0,0	100,0
Medicações Uso Contínuo	64,5	36,2	80,0	10,0	100,0
Histórico Cirúrgico	15,5	24,2	0,0	0,0	70,0
Isolamentos ou Precauções	25,5	34,2	10,0	0,0	90,0
Barreiras para Comunicação	80,0	33,5	100,0	10,0	100,0
**Avaliação**					
Aparência das Mucosas	8,2	21,4	0,0	0,0	70,0
Nível de Consciência	73,6	41,5	100,0	0,0	100,0
Locomoção	74,5	38,3	100,0	0,0	100,0
Sistema Oxigenação	46,4	42,7	20,0	0,0	100,0
Cateteres Venosos	78,2	23,6	80,0	30,0	100,0
Presenças de Drenos	29,1	20,2	30,0	0,0	70,0
Vias Administração Medicações	81,8	28,9	100,0	10,0	100,0
Esquema Nutricional	60,9	37,5	70,0	0,0	100,0
Curativos, Incisões	38,2	21,8	40,0	0,0	80,0
Presença de Estomias	11,8	15,4	10,0	0,0	40,0
Sinais Vitais	70,0	36,3	90,0	0,0	100,0
Presença de Dor	49,1	25,1	60,0	0,0	80,0
Hemoglicoteste	40,9	29,8	50,0	0,0	100,0
Eliminações Fisiológicas	74,5	35,3	90,0	10,0	100,0
Medicções Especias	60,0	22,8	50,0	30,0	100,0
**Recomendação**					
Interconsultas de Enfermagem	0,0	0,0	0,0	0,0	0,0
Intervenções de Enfermagem	0,0	0,0	0,0	0,0	0,0
Outros/Intercorrências	11,8	21,4	0,0	0,0	70,0
Assinatura	42,7	49,4	0,0	0,0	100,0

A tabela 7 demonstra os itens avaliados pelo conjunto de itens das variáveis: característica do paciente; riscos identificados, situação, breve

história, avaliação e recomendação. Evidenciando as variáveis mais e menos preenchidas no instrumento.

Tabela 7. Dados das variáveis preenchidas no instrumento de avaliação da passagem de plantão pelos participantes. Botucatu, 2019.

Variáveis	media	± dp	mediana (min-max)
**Característica do paciente**	82,7	± 23,3	90,0 (18,2 - 100,0)
**Riscos identificados**	46,9	± 38,4	66,8 (0,0 - 81,8)
**Situação**	59,6	± 38,3	78,2 (0,0 - 90,0)
**Breve história**	50,3	± 25,2	57,3 (15,5 - 80,0)
**Avaliação**	50,3	± 27,7	53,6 (8,2 - 81,8)
**Recomendação**	13,6	± 20,2	5,9 (0,0 - 42,7)

Para ilustrar esses achados o quadro 4 descreve os itens do instrumento considerando a ordem do mais preenchido para o menos preenchido.

Quadro 4. Itens por ordem de preenchimento.

Ítem	%
Nome	100,0
Leito	94,5
Data do Dia	92,7
RG	90,9
Data da Admissão	90,0
Sexo	90,0
Diagnóstico Médico	90,0
Especialidade	87,3
Idade	85,5
Data de Nascimento	83,6
Dia de Internação	81,8
Acompanhante	81,8
Vias Administração Medicações	81,8
Visita Familiar	80,9
Identificação (pulseira)	80,0
Placa de Leito	80,0
Barreiras para Comunicação	80,0
Pré ou Pós Operatório, Clínico	78,2
Cateteres Venosos	78,2
Lesão por Pressão	77,3
Locomoção	74,5
Eliminações Fisiológicas	74,5
Nível de Consciência	73,6
Sinais Vitais	70,0
Alergias	66,4
Medicações Uso Contínuo	64,5

Esquema Nutricional	60,9
Medicações Especiais	60,0
Higiene e Conforto	56,4
Co-morbidades	50,0
Presença de Dor	49,1
Sistema Oxigenação	46,4
Médico Responsável	42,7
Assinatura	42,7
Hemoglicoteste	40,9
Curativos , Incisões	38,2
Presenças de Drenos	29,1
Isolamentos ou Precauções	25,5
Religião	18,2
Histórico Cirúrgico	15,5
Presença de Estomias	11,8
Outros	11,8
Risco de Queda	10,9
Aparência das Mucosas	8,2
Protocolo de TEV	0,9
Escala de Fugulin	0,9
Qualidade de Assitência	0,0
Diagnosticos de Enfermagem	0,0
Interconsultas de Enfermagem	0,0
Intervenções de Enfermagem	0,0

7- DISCUSSÃO

O presente estudo teve como objetivo principal descrever o processo de construção e validação semântica de um instrumento de passagem de plantão usando a metodologia *SBAR*, disponibilizando um impresso prático e fácil de usar, auxiliando profissionais que utilizam a passagem de plantão como instrumento de trabalho diário, essa ferramenta visa auxiliar e melhorar a comunicação entre as equipes de saúde.

Para a etapa da construção do instrumento foi realizada uma revisão da literatura sobre instrumentos de passagem de plantão e, conforme a realidade da enfermaria de gastroenterologia cirúrgica do hospital cenário do estudo, a primeira versão do instrumento continha os itens de identificação do paciente; indicadores; Situação (S); Breve história (B); Avaliação (A) e Recomendação (R).

A construção do instrumento está de acordo com estudo realizado por Cornell et al que desenvolveram um formulário eletrônico de passagem de plantão utilizando a ferramenta SBAR, incluindo os elementos do cuidado de enfermagem. Os autores demonstraram que o instrumento possibilitou a comunicação dos profissionais de enfermagem de forma eficiente e com aproveitamento do tempo, pois a utilização do SBAR permitiu foco, consistência de informações e promoção do diálogo entre a equipe de enfermagem.

Estudiosos como Corpolato[37] corrobora a necessidade de construção de instrumentos de passagem de plantão, pois considera que o instrumento auxilia na transmissão de informações durante esse processo e contribui para garantir a segurança do paciente minimizando os riscos e falhas na comunicação.

A primeira versão do instrumento construído, foi avaliada por dez juízes, enfermeiros especialistas e experientes na atuação clínica. Esses juízes eram predominantemente do sexo feminino, com idade média de 42,5 anos, com média de exercício profissional de 16 anos, atuando como docentes, especialistas e doutores. A seleção dos juízes considerou a experiência e a qualificação desses membros. A seleção de especialistas para a avaliação de instrumentos é tão importante quanto à definição dos domínios do instrumento, pois um método sistematizado de julgamento de informações, utilizado para obter consenso de especialistas sobre determinado tema para obter-se validação é o que se propõe com a técnica Delphi.[38]

As etapas de construção e validação do instrumento foram realizadas com base na metodologia sugerida por Pasquali[36] sendo: elaboração de itens do instrumento, avaliação pelo Comitê de Juízes, análise semântica e a realização do piloto, para tal, esse modelo foi o mais adequado para a construção do instrumento.

Após análise dos juízes e mesmo obtendo um IVC de 91.7%, os itens do instrumento tiveram sugestões e ao analisar-se as mesmas, incluiu-se e excluiu-se aquelas que estavam de acordo com a proposta da ferramenta SBAR. Feita as adequações, o instrumento modificado incluiu no item identificação o número de registro, dia de internação e religião; o item indicadores foi substituído por riscos identificados e nesse item incluíram-se: higiene e conforto; tipo de cuidado com as alternativas: banho no leito, banho de cadeira, banho de aspersão com e sem auxílio; sistema de classificação de pacientes, qualidade de assistência e visita familiar, para todas essas inclusões as alternativas para preenchimento das lacunas foi de sim ou não. No item Situação(S) forma incluídos: especialidade médica responsável pelo paciente, a condição do paciente sendo pré ou pós operatório e diagnóstico médico atual. Em Breve história(B) nada foi sugerido. Em Avaliação(A) incluiu-se no campo medicamentos as vias de administração mais utilizadas em formato de *checklist*, fármacos de controle especial, escala de dor e datas das trocas de drenos e sondas. Nesse item foi excluído exames laboratoriais. No item Recomendação (R) foi substituído interconsulta por interconsultas de enfermagem e intercorrências.

A inclusão e exclusão dos itens referidos acima, possibilitou a construção da segunda versão do instrumento, considerando-a como versão final, a ser aplicada na passagem de plantão da enfermaria de gastroenterologia cirúrgica e analisada pelos técnicos de enfermagem dessa enfermaria.

O formato de *checklist* foi considerado adequado pelos juízes sendo corroborado pela literatura que ressalta ser esta modalidade facilitadora do raciocínio clínico e das informações dos pacientes.[37 39]

Os itens que compuseram o instrumento estão de acordo com o proposto pela Organização Mundial da Saúde (OMS) que destaca ser a padronização uma importante ferramenta de comunicação e a ferramenta SBAR um formato adequado para essa comunicação.[3]

Nesse estudo, os juízes puderam opinar em relação à clareza, pertinência e aparência do instrumento, redigindo suas sugestões de inclusão e exclusão, ou seja, modificando os itens através de comentários em campo destinado para esse fim. Com intuito de verificar repetidas vezes as adaptações semânticas, Beaton,[40] descrevem como imprescindível a inclusão de mais de uma avaliação pelo comitê de Juízes.

Após a validação de face e de conteúdo, o instrumento passou pela análise semântica com uma amostra de 11 participantes, técnicos de enfermagem que compõem o quadro funcional do cenário do estudo com predominância do sexo feminino, idade média de 37,9 anos, atuação profissional de 10,3 anos.

Esse perfil de profissional está de acordo com a pesquisa realizada em nível nacional sobre a enfermagem brasileira. No estado de São Paulo, 71,1% dos profissionais são técnicos ou auxiliares de enfermagem, 82,3% do sexo feminino na faixa etária de 26 a 40 anos (58,7%).[41]

O teste piloto foi implantado e os participantes aplicaram 351 instrumentos de passagem de plantão no mês de fevereiro de 2019. De acordo com estudos anteriores, essa fase faz-se necessário, tendo em vista o objetivo de verificar se o instrumento ficou claro e compreensivo aos membros que farão uso do mesmo.[42 43 44]

Realizados os testes, os participantes responderam ao questionário referente ao instrumento e a ferramenta SBAR.

Os participantes referiram que a passagem de plantão é predominantemente oral, ocorre na sala da enfermagem em um tempo de seis a dez minutos com o colega prestando atenção. No entanto, quando questionados sobre as interferências na passagem de plantão destacaram os atrasos, as saídas antecipadas e as conversas paralelas dos colegas, sendo essa afirmação contraditória a anterior. Infere-se que os participantes responderam inicialmente o que seria ideal no processo de passagem de plantão e posteriormente o que de fato ocorre.

A modalidade oral de passagem de plantão é corroborada em estudo de Gonçalves.[9]

Contrariamente ao presente estudo, o local para a passagem de plantão proposto na literatura foi a modalidade à beira leito, pois é uma forma de

promoção da segurança nos serviços de saúde, possibilitando a participação do paciente em seu cuidado.[9 37 45]

Quanto ao tempo para a passagem de plantão a literatura ressalta ser suficiente o de dez a 20 minutos diferentemente do encontrado no presente estudo. Os autores referem que o tempo dispendido na passagem de plantão será determinante na quantidade e qualidade das informações.[9 37]

A literatura expressa que os enfermeiros reclamam sobre o tempo prolongado para a passagem de plantão e destaca que a passagem à beira leito diminui o tempo evitando as horas extras em consequência de atrasos na saída da equipe de enfermagem.[15 45]

Dos fatores que interferem negativamente na passagem de plantão, a literatura ressalta serem as interrupções, ruídos externos, impontualidade e conversas paralelas entre os membros da equipe os mais frequentes.[9 37]

Dentre os questionamentos, ressalta-se que 100% deles consideraram que o instrumento embasado na metodologia SBAR possui 100% das informações necessárias para a passagem de plantão, a maioria avaliou o instrumento como bom e muito bom. Também referem ser o instrumento viável para ser implantado na unidade.

Estudo, com objetivo de implementar uma ferramenta de passagem de plantão por meio do acrônimo ISBAR (*Identification, Situation, Background, Assessment & Action, Response/Rationale*) em uma unidade de pronto atendimento concluiu que é crucial utilizar uma ferramenta padrão, formal e sistematizada para garantir que a transferência dos cuidados seja “eficaz, completa e objetiva” com possibilidade da segurança do paciente e equipe.[46]

Estudos internacionais ressaltam que o uso da ferramenta SBAR para a passagem de plantão é uma forma padronizada eficaz de padronização da comunicação entre os membros da equipe de enfermagem, além de ser benéfica aos pacientes, contribuindo para a satisfação da equipe e segurança do paciente.[1 2 30 31 32]

Na aplicação do instrumento no cenário da pesquisa, os participantes foram técnicos de enfermagem, sendo analisados 110 instrumentos dos 351 aplicados, considerando-se dez instrumentos de cada participante e o período de aplicação (início, meio e fim do mês selecionado). Essa estratégia visou

identificar se houve mudança no preenchimento ao longo do tempo, sendo que essa hipótese não foi confirmada.

A opção de trabalhar apenas com os técnicos de enfermagem foi em virtude desses realizarem a passagem de plantão e os enfermeiros terem sido juízes do instrumento, pois eram os especialistas na área de gastroenterologia cirúrgica, unidade que foi escolhida como cenário do estudo.

Assim, a análise de preenchimento do instrumento evidenciou que os itens: diagnóstico de enfermagem, interconsultas de enfermagem, intervenção de enfermagem e qualidade de assistência não tiveram nenhum preenchimento, pois consideram-se atividades privativas dos enfermeiros. Outros com preenchimento mínimo (abaixo de 10%) foram a aparência das mucosas, Escala de classificação de pacientes, segundo Fugulin, protocolo de tromboembolismo venoso (TEV), que também são atividades exclusivas dos enfermeiros.

A Lei 7.498/86 que dispõe sobre a regulamentação do exercício da enfermagem, no artigo 11, refere ser privativo do enfermeiro as atividades de “planejamento, organização, coordenação, execução e avaliação dos serviços da assistência de enfermagem”, além de “consultoria, auditoria e emissão de parecer sobre matéria de enfermagem”, “consulta de enfermagem” e “prescrição da assistência de enfermagem” [47], possibilitando compreender que o técnico de enfermagem conhece as suas atribuições.

Outros itens com baixo preenchimento (menores que 50%) foram os referentes a: presença de dor, oxigenação, médico responsável, assinatura, curativos e incisões, presença de drenos ou precauções, religião do paciente, riscos de queda, histórico cirúrgico e presença de ostomias. Infere-se que esses profissionais não consideram esses itens indispensáveis para a comunicação entre a equipe, sendo necessário investir no processo educativo da equipe sobre a importância desses itens para garantir a satisfação e segurança do paciente.

Estudos anteriores ressaltam que a sistematização e padronização da passagem de plantão possibilitam a resolução de problemas e riscos relacionados à falta de comunicação e, como consequência, reduzem os eventos adversos. [37]

Os itens mais valorizados no preenchimento referem-se as características do paciente e riscos identificados.

Estudos demonstram que a adequada identificação do paciente e dos riscos são fundamentais para evitar eventos adversos.[13] [1]

8 - CONCLUSÃO

De acordo com a proposta, o instrumento construído foi validado semanticamente e sua aplicação evidenciou a relevância, pois considera-se o instrumento necessário e viável. Os itens com maior preenchimento foram relativos a característica do paciente sendo a identificação o item mais preenchido e o item recomendação teve menor preenchimento, pois como técnicos de enfermagem consideram que alguns itens dessa variável são privativos do enfermeiro.

Este estudo contribuiu para o ensino e pesquisa em enfermagem, descrevendo cada etapa de construção de um instrumento, dessa forma, com a construção, validação semântica e os resultados encontrados de forma positiva, torna o instrumento foco desse estudo, em plena condição de ser aplicado na prática, o que possibilitará uma comunicação efetiva entre as equipes de saúde.

A limitação do estudo refere-se a não inclusão de enfermeiros na aplicação do instrumento e o único cenário da atenção hospitalar.

Para implementação do instrumento recomenda-se que seja realizada uma padronização informatizada para a passagem de plantão, tendo em vista que a informatização ampliaria a possibilidade do registro das informações relevantes, favoreceria a continuidade da assistência, otimizaria o tempo de preenchimento, garantindo a segurança do paciente e o respaldo ético e legal do profissional.

REFERÊNCIAS

1. Cornell P, Gervis MT, Yates L, Vardaman JM. Impact of SBAR on nurse shift reports and staff rouding. Med. Surg Nursing. 2014, 23(5):334-42.

2. Cornell P, Gervis MT, Yates L, Vardaman JM.Improving shift report focus and consistency with the Situation, Background, Assessment, Recommendation protocol. JONA. 2013, 43(7/8):422-8.

3. World Health Organization (WHO). World alliance for patient safety. Communication during patient hand-overs. Patient safety solutions [Internet]. 2007 [acesso 2019 Mar 08]. Available from: https://www.who.int/patientsafety/solutions/patientsafety/PS-Solution3.pdf?ua=1

4. Silva EE, Campos LF. Passagem de plantão na enfermagem: revisão de literatura. Cogitare Enferm 2007 Out/Dez; 12(4):502-7.

5. Pinho DLM, Abrahão JI, Ferreira MC. As estratégias operatorias e a gestão da informação no trabalho de enfermagem no contexto hospitalar. Revista latino – Am. Enferm, 2003, 11(2):168-176.

6. Portal KM, Magalhães AMM. Passagem de plantão: um recurso estratégico para a continuidade do cuidado em enfermagem. Rev. Gaúcha Enferm., Porto Alegre (RS) 2008 jun;246-53.

7. Siqueira ILCP, Kurcgant P. Passagem de plantão: falando de paradigmas e estratégias. Acta Paul Enferm. 2005;18(4):446-51.

8. Marques LF, Santiago LC, Felix VC. Passagem de plantão realizada pela equipe de enfermagem em um hospital público de ensino. Journal of Nursing UFPE, 2012, 6(9):2052-7. Doi: 10.5205/reuol.2570-20440-1-LE.0609201207

9. Gonçalves MI, Rocha PK, Anders JC, Kusahara DM, Tomazoni A. Comunicação e segurança do paciente na passagem de plantão em unidades de cuidados intensivos neonatais. Texto & Contexto Enferm. 2016; 25(1):e2310014.

10. Conselho Federal de Enfermagem. Resolução COFEN Nº 564/2017. Disponível em: http://www.cofen.gov.br/resolucao-cofen-no-5642017_59145.html

11. Silva NDM, Barbosa AP, Kátia Grillo Padilha, Malik AM. Segurança do paciente na cultura organizacional: percepção das lideranças de instituições hospitalares de diferentes naturezas administrativas. Rev Esc Enferm USP. 2016;50(3):490-497.

12. Konh LT; Corrigan JM; Donaldson MS. Errors in health care: a leanding cause of death and injury. In: Konh LT, Corrigan JM, Donaldson MS. To

Err is Human: building a safer health care system [Internet]. Whashington (DC): Institute of Medicine; 2000 [cited 2015 Oct 20]. P.26-48. Available from: http: //www.nap.edu/openbook.php?record_id=9728&Page=R

13. Caldana G, Guiardello EB, Urbanetto JS, Peterlini MAS, Gabriel CS. Brazilian network for nursing and patient safety: challenges and perspectives. Texto Contexto Enferm [Internet]. 2015; 24(3):906-11.

14. Agência Nacional de Vigilância Sanitária (BR). Segurança do paciente e qualidade em serviços de saúde: uma reflexão teórica aplicada à prática. Cartilha. Brasília (DF): ANVISA; 2013.

15. Pontes EP, Couto DL, Lara HMS, Santana JCB. Nonverbal communication in the pediatric intensive care unit: perception of the multidisciplinary team. Rev Min Enferm. 2014; 18(1):158-63.

16. Welsh CA., Flanagan ME, Ebright P. Barriers and facilitators to nursing handoffs: Recommendations for redesign. Nursing Outlook [Internet]. 2010 [cited 2015 Jun 10]; 58(3):. Available from: http:// www.sciencedirect.com/science/article/pii/ S002965540900195X.

17. Ministério da Saúde (Brasil) Secretaria de Atenção à Saúde, Núcleo Técnico da Política Nacional de Humanização. Humaniza SUS: documento base para gestores e trabalhadores do SUS. 4. ed. Brasília: Ministério da Saúde, 2008.

18. Barra DCC, Waterkemper R, Kempfer SS, Carraro TE, Radünz V. Hospitalidade como expressão do cuidado em enfermagem. Rev Bras Enferm 2010;63(2):203-8.

19. Backes DS, Lunardi VL, Lunardi FWD. A humanização hospitalar como expressão da ética. Rev Latinoam Enferm 2006;14(1):132-5.

20. Stefanelli MC, Carvalho EC (orgs.) A comunicação nos diferentes contextos da enfermagem. Barueri, SP, Manole, 2005.

21. Silva MJP. Comunicação tem remédio: a comunicação nas relações interpessoais em saúde. São Paulo, Loyola, 2012.

22. Barlem ELD, Rosenhein DPN, Lunardi VL, Lunardi Filho WD. Comunicação como instrumento de humanização do cuidado de enfermagem: experiências em unidade de terapia intensiva. Rev. Eletr. Enf. [periódico online]. 2008;10(4):1041-9. Disponível em: . Acesso em junho de 2018.

23. National Institute for Health Research. Patient safety 2030. 2016. Available from: https://www.imperial.ac.uk/media/imperial-college/institute-of-global-health-innovation/centre-for-health-policy/Patient-Safety-2030-Report-VFinal.pdf

24. Braga, EM, Silva MJP. Comunicação competente: visão de enfermeiros especialistas em comunicação**.** *Acta paul. enferm.* [online]. 2007, vol.20, n.4, pp.410-414.

25. Gonçalves MI, Rocha PK, Souza S, Tomazoni A, Paz BPD, Souza AIJ. Segurança do paciente e passagem de plantão em cuidados intensivos neonatais. Rev. Baiana enferm. 2017; 31(2):e17053.

26. Manser T, Foster S, Gisin S, Jaeckel D, Ummenhofer W. Assessing the quality of patient handoffs at care transitions. Qual Saf Health Care [Internet]. 2010; 19(6). Available from: http:// qualitysafety.bmj.com/content/19/6/1.55.full. pdf+html. Acesso em julho 2018. Medicina Net . Acesso em abril de 2018.

27. Holly C, Poletick EB. A systematic review on the transfer of information during nurse transitions in care. J Clin Nurs [Internet]. 2014; 23(17-18):2387-95. Available from: http:// onlinelibrary.wiley.com/doi/10.1111/jocn.12365/full 10.

28. Institute for Healthcare Improvement . Acesso em abril de 2018.

29. Manning, M.L., 2006. Improving clinical conversations through structured conversations. Nurse Econ. 24 95), 268-271.

30. Ting WH, Peng FS, Lin HH, Hsiao SM. The impact of situation-background-assessment-reommendation (SBAR) on safety attitudes in the obstetrics department. Taiwanese Journal of Obstetrics & Gynecology. 2017; 56:171-4.

31. Martin HA, Ciurzynski SM. Situation, background, assessment, and recommendation – guide huddles improve communication and teamwork in the emergency department. Journal of Emergency Nursing. 2015;41(6):484-8.

32. Stewart KR, Hand KA. SBAR, communication, and patient safety: an integrated literature review. Medsurg Nursing. 2017; 26(5):297-305.

33. Polit D. F; Beck C. T. Fundamentos de Pesquisa em enfermagem: avaliação de evidências para as práticas da enfermagem. 7a ed. Porto Alegre (RS): Artmed; 2011. 669 p.

34. Lobiondo – Wood G; Haber J. Nursing Research: Methods and Critical Appraisal for Evidence – Based Practice. In: ___. 8ºed. Philadelphia: Mosby Elsevier, 2013. Cap. Nonexpermental designs, p.199.

35. Hospital das Clínicas – Faculdade de Medicina de Botucatu. História, os primórdios do HC. Disponível em: <>. Acesso em março de 2019.

36. Pasquali L. Instrumentação Psicológica: Fundamentos e Práticas. 1º ed. Porto Alegre: Artmed, 2010. 560 p.

37. Corpolato RC, Mantovani MF, Willig MH, Andrade LAS de, Mattei AT, Arthur JP. Padronização da passagem de plantão em Unidade de Terapia Intensiva Geral Adulto. Rev. Bras. Enferm. [Internet]. 2019 [citado 2019 Jun 24]; 72(Suppl 1): 88-95. Disponível em: http://www.scielo.br/scielo.php?script=sci_arttext&pid=S0034-71672019000700088&lng=pt. http://dx.doi.org/10.1590/0034-7167-2017-0745.

38. Scarparo AF, Laus AM, Azevedo ALCS, Freitas MRI, Gabriel CS, Chaves, LDP. Reflexões sobre o uso da técnica Delphi em pesquisas na enfermagem. Rev Rene [en linea] 2012, 13 [Acesso em: 24 de junio de 2019] Disponível em:**<http://www.redalyc.org/articulo.oa?id=324027980026>**

39. Ramalho Neto JM, Fontes WD de, Nóbrega MML da. Instrumento de coleta de dados de enfermagem em Unidade de Terapia Intensiva Geral. Rev. bras. enferm. [Internet]. 2013 [citado 2019 Jun 24];66(4):535-42. Disponível em: http://www.scielo.br/scielo.php?script=sci_arttext&pid=S0034-71672013000400011&lng=pt. http://dx.doi.org/10.1590/S0034-71672013000400011.

40. Beaton, D. S. et al. Guidelines for the process off cross cultural adaptation of self report measures. Spine, Hagerstown, v. 25, n. 24, p. 3186-3191, 2000.

41. Conselho Federal de Enfermagem – COFEN. Pesquisa do Perfil dos Profissionais de Enfermagem no Brasil [Internet]. Brasília: COFEN; 2015 [citado 24 Jun 2019]. Disponível em: http://www.cofen.gov.br/perfilenfermagem/

42. Junior, M. S. B.; Matsuda, L. M. Construção e validação de instrument para avaliação do acolhimento com classificação de risco. Revista Brasileira de Enfermagem, Brasília, v65, n. 5, p. 751 – 757, 2012.

43. Almeida, C. M. T.; Rodrigues, V. M. C. P.; Escola, J. J. J. The representations of human vulnerability held by health workers – development and validation of a scale. Rev. Latino-Am. Enfermagem, Ribeirão Preto, v 21, n SPE, p. 29-37, 2013.

44. Oliviera, A.P.A.; Nascimento, E. Construção de uma escala para avaliação do planejamento cognitivo. Psicologia Reflexão e Crítica, Porto Alegre, v. 27, n 2, p. 209-2018, 2014.

45. Novak K., Fairchild R. Bedside reporting and SBAR: improving patient communication and satisfaction. Journal of Pediatric Nursing. 2012;27:760-2. http://dx.doi.org//10.1016/j.pedn

46. Silva DA, Rocha IMS, Dias FA, Moreira DA, Afonso LN, Brito MJM. Otimização da ferramenta utilizada durante a passagem de plantão em uma unidade de pronto atendimento. SANARE [Internet]. 2017 [cited 2019 Jul 11];16(1):118-23. Available from: https://sanare.emnuvens.com.br/sanare/article/view/1102

47. Brasil, Lei N 7.498/86, de 25 de junho de 1986 que dispõe sobre a regulamentação do exercício da Enfermagem e dá outras providências. Disponível em: http://www.cofen.gov.br/lei-n-749886-de-25-de-junho-de-1986_4161.html.

APÊNDICE 1

TERMO DE CONSENTIMENTO LIVRE E ESCLARECIDO (TCLE)

RESOLUÇÃO 466/2012

CONVIDO, o Senhor (a) para participar do Projeto de Pesquisa intitulado **"Construção e Validação Semântica de um Instrumento de Passagem de Plantão"**, que será desenvolvido por mim, Tânia Roberta Limeira Felipe aluna do Mestrado Profissional de Enfermagem, com orientação da Enfermeira e Professora Dra. Wilza Carla Spiri, da Faculdade de Medicina de Botucatu – UNESP.

Este estudo tem como finalidade construir um instrumento de passagem de plantão por meio da ferramenta *SBAR* (ferramenta essa que possibilita uma melhor comunicação entre profissionais de saúde) pois acredita ser esta, necessária para melhorar a qualidade da informação transmitida entre os turnos e até mesmo entre os setores, como por exemplo, na transferência de pacientes, resultando na continuidade da assistência.

Os benefícios de sua participação no estudo é possibilitar que a ferramenta *SBAR* seja aprimorada para a passagem de plantão e implementada em instituições favorecendo a comunicação entre os profissionais de Enfermagem com consequente melhoria na qualidade do cuidado e na segurança do paciente.

Fique ciente de que sua participação neste estudo é voluntária e que mesmo após ter dado seu consentimento para participar da pesquisa, você poderá retirá-lo a qualquer momento.

Este Termo de Consentimento Livre e Esclarecido será elaborado em 2 vias de igual teor, o qual 01 via será entregue ao Senhor (a) devidamente rubricada, e a outra via será arquivada e mantida pelos pesquisadores por um período de 5 anos após o término da pesquisa.

Qualquer dúvida adicional você poderá entrar em contrato com o Comitê de Ética em Pesquisa através dos telefones (14) 3880-1608 ou 3880-1609 que funciona de 2ª a 6ª feira das 8.00 às 11.30 e das 14.00 às 17horas, na Chácara Butignolli s/nº em Rubião Júnior – Botucatu - São Paulo. Os dados de localização dos pesquisadores estão abaixo descrito:

Após terem sido sanadas todas minhas dúvidas a respeito deste estudo, CONCORDO EM PARTICIPAR de forma voluntária, estando ciente que todos os meus dados estarão resguardados através do sigilo que os pesquisadores se comprometeram. Estou ciente que os resultados desse estudo poderão ser publicados em revistas científicas, sem, no entanto, que minha identidade seja revelada.

Botucatu,_____/___/______

________________________ ________________________

Pesquisador Participante da Pesquisa

Pesquisadora: Tânia Roberta Limeira Felipe
Endereço: Rua Vitório Brollo, 31 – CEP 18.650-000 São Manuel /SP
Telefone: (14) 991163818 E-mail: enf.tania2013@gmail.com

Orientadora: Profª Drª Wilza Carla Spiri
Endereço: Departamento de Enfermagem – Faculdade de Medicina – Unesp – Campus Rubião Junior SN – CEP 18.618-970 Botucatu/ SP
Telefone: (14) 38801320 – E-mail: wilza@fmb.unesp.br

APÊNDICE 2

AVALIAÇÃO DOS JUÍZES

Caro juiz, solicito a sua análise do instrumento de passagem de plantão elaborado que será utilizado pelos profissionais de Enfermagem de uma unidade clínica hospitalar. Por favor, preencha os dados de caracterização e a análise (concordo/discordo) necessita ser realizada em relação a **clareza**, **pertinência** e **aparência**, considerando os seguintes conceitos:

- **Clareza**: atributo daquilo que é inteligível; fácil de ser compreendido ou entendido.
- **Pertinência:** característica do que é concernente, apropriado e relevante.
- **Aparência:** é o aspecto ou aquilo que se mostra superficialmente ou à primeira vista.

Para os itens em que houver discordância haverá espaço para realizar suas considerações. Ao final do instrumento também há disponível um espaço para sugestões adicionais, que podem não estar ligadas a um item específico.

CARACTERIZAÇÃO:

Idade: _________ (anos)   Sexo: Feminino ( ) Masculino ( )

Atuação Profissional: Gestão ( ) Assistência ( ) Docência ( ) Outros ( ) ____ _______________

Tempo de Atuação Profissional ______ (anos)

Formação:

Somente graduação ( ) Especialização ( ) Mestrado ( ) Doutorado ( ) Pós Doutorado ( )

ANÁLISE DO INSTRUMENTO

Dados de Identificação:

Clareza: Concordo ( ) Discordo ( )

Pertinência: Concordo ( ) Discordo ( )

Aparência: Concordo ( ) Discordo ( )

Tenho as seguintes sugestões: ____
____
____
____
____

Indicadores:

Clareza: Concordo ( ) Discordo ( )

Pertinência: Concordo ( ) Discordo ( )

Aparência: Concordo ( ) Discordo ( )

Tenho as seguintes sugestões: ____
____
____
____
____

(S) Situação:
Clareza: Concordo ( ) Discordo ( )
Pertinência: Concordo ( ) Discordo ( )
Aparência: Concordo ( ) Discordo ( )
Tenho as seguintes sugestões:
____
____
____
____
____

(B) Breve História:
Clareza: Concordo ( ) Discordo ( )
Pertinência: Concordo ( ) Discordo ( )
Aparência: Concordo ( ) Discordo ( )
Tenho as seguintes sugestões:
____
____
____
____
____

(A) Avaliação:
Clareza: Concordo ( ) Discordo ( )
Pertinência: Concordo ( ) Discordo ( )
Aparência: Concordo ( ) Discordo ( )
Tenho as seguintes sugestões:
____
____
____

(R) Recomendação:
Clareza: Concordo ( ) Discordo ( )
Pertinência: Concordo ( ) Discordo ( )
Aparência: Concordo ( ) Discordo ( )
Tenho as seguintes sugestões:
____
____
____
____
____

Outras Sugestões:
____
____
____
____

APÊNDICE 3 – QUESTIONÁRIOS PARA OS PARTICIPANTES DO ESTUDO

AVALIAÇÃO DOS PARTICIPANTES

Caro participante, esse momento da pesquisa necessita de algumas informações adicionais e a sua avaliação ao instrumento elaborado por mim (o mesmo que você utilizou para passagem de plantão), par que o estudo siga adiante. Ao final do questionário, haverá um campo para sugestões adicionais.

CARACTERIZAÇÃO

Participante Nº: _________

Idade: _________ Sexo: ( ) Feminino ( ) Masculino

Tempo de Atuação Profissional: ___________

Formação: ( ) Nível Técnico ( ) Nível Superior

INFORMAÇÕES ADICIONAIS

1 – Modalidade utilizada para passagem de plantão.

( ) Verbal ( ) Escrita ( ) Verbal e escrita

2 – Local onde ocorre a passagem de plantão.

( ) Ao lado do paciente ( ) No corredor da unidade ( ) Sala de enfermagem ( ) Outro

3 – Tempo utilizado para a passagem de plantão.

( ) até 5 min. ( ) 6 a 10 min. ( ) 11 a 20 min. ( ) 21 a 30 min. ( ) 31 min. ou mais

4 – Registro de informações sobre o paciente no livro de ocorrência e/ou prontuário.

( ) Sim ( ) Não

5 – O momento da passagem de plantão é um momento oportuno para questionamentos e esclarecimentos de dúvidas.

( ) Sim ( ) Não

6 - Comportamento do colega durante a passagem de plantão.

a) Presta atenção às informações.

( ) Sim ( ) Não

b) Conversas paralelas

( ) Sim ( ) Não

c) Atrasos

( ) Sim ( ) Não

d) Realiza procedimentos durante a passagem de plantão.

( ) Sim ( ) Não

7 – Atrasos e saídas antecipadas interferem na passagem de plantão.

( ) Sim ( ) Não

8 – As interferências na passagem de plantão ocorrem devido:

( ) Interrupções ( ) Conversas paralelas ( ) Ruídos ( ) Questionamentos

( ) Necessidade de repetir a informação ( ) Outros

<u>AVALIAÇÃO DO INSTRUMENTO SBAR</u>

1 – O instrumento SBAR possui as informações necessárias.

( ) Sim ( ) Não

2 – Como você caracteriza a passagem de plantão com o uso da ferramenta SBAR.

( ) Ruim ( ) Regular ( ) Boa ( ) Muito boa ( ) Excelente

3 – São informações necessárias para passagem de plantão.

( ) Intercorrências ( ) Condição clínica ( ) Questões administrativas ( ) Exames

( ) Medicações ( ) Alterações no tratamento ( ) Identificação do paciente

( ) Família e acompanhante ( ) Outros

4 – Acha viável implantar o SBAR na unidade.

( ) Sim ( ) Não

SUGESTÕES:

____

____

____

____

____

____

ANEXO 1- Aprovação do Comitê de Ética em Pesquisa

UNESP -FACULDADE DE MEDICINA DE BOTUCATU

PARECER CONSUBSTANCIADO DO CEP

DADOS DO PROJETO DE PESQUISA

Título da Pesquisa: CONSTRUÇÃO E VALIDAÇÃO SEMÂNTICA DE UM INSTRUMENTO DE PASSAGEM DE PLANTÃO

Pesquisador: TANIA ROBERTA LIMEIRA FELIPE

Área Temática:

Versão: 1

CAAE: 02168918.0.0000.5411

Instituição Proponente: Departamento de Enfermagem

Patrocinador Principal: Financiamento Próprio

DADOS DO PARECER

Número do Parecer: 3.009.068

Apresentação do Projeto:

Trata-se de pesquisa qualitativa, a partir da construção de um instrumento de passagem de plantão considerando a metodologia SBAR e aplicado aos juízes para a validação semântica.

Método: Trata-se de estudo metodológico cujo objetivo é trabalhar com instrumentos e ferramentas complexas e desenvolvimento de referenciais metodológicos. A proposta será construir e validar semanticamente uma passagem de plantão padronizada. Os enfermeiros assistenciais farão a avaliação. A pesquisa metodológica aborda o desenvolvimento e validação do instrumento em uma determinada área de assistência de enfermagem. Esse tipo de estudo abrange quatro etapas, a saber: definição do conceito ou comportamento a ser medido, formulação dos itens do instrumento, desenvolvimento de instruções para o teste de confiabilidade e da validade do instrumento de medida.

Tamanho amostral: 10 participantes.

Objetivo da Pesquisa:

Objetivo Primário: Descrever o processo de validação semântica de um instrumento de passagem de plantão utilizando a metodologia SBAR.

Avaliação dos Riscos e Benefícios:

Riscos: por se tratar de pesquisa de opinião de especialistas, poderá ocorrer constrangimento do

participante envolvido no estudo, no que diz respeito duvidas às respostas, que serão minimizadas por sigilo e privacidade.
Benefícios: A pesquisa possibilitará que a ferramenta SBAR seja aprimorada para a passagem de plantão e implementada em instituições favorecendo a comunicação entre os profissionais de Enfermagem com consequente melhoria na qualidade do cuidado e na segurança do paciente.

Comentários e Considerações sobre a Pesquisa:

Trata-se de pesquisa relevante e adequadamente elaborada. O pesquisador informa que haverá um custo de R$ 500,00, com financiamento próprio.

Considerações sobre os Termos de apresentação obrigatória:

Documentação adequada, foram apresentados todos os termos obrigatórios. TCLE adequado e, em forma de convite, de acordo com a Resolução n. 466/2012.

Recomendações:

Não há.

Conclusões ou Pendências e Lista de Inadequações:

Após análise, o Colegiado deliberou APROVADO o projeto de pesquisa apresentado.

Considerações Finais a critério do CEP:

Conforme deliberação do Colegiado em reunião ordinária do Comitê de Ética em Pesquisa da FMB/UNESP, realizada em 05 de novembro de 2018, o projeto encontra-se APROVADO, sem (com) necessidade de envio à CONEP.
No entanto, informamos que ao final da execução da pesquisa, seja enviado o "Relatório Final de Atividades", na forma de "Notificação", via sistema Plataforma Brasil.
Atenciosamente,
Comitê de Ética em Pesquisa da Faculdade de Medicina de Botucatu – UNESP

Este parecer foi elaborado baseado nos documentos abaixo relacionados:

Tipo Documento	Arquivo	Postagem	Autor	Situação
Informações Básicas do Projeto	PB_INFORMAÇÕES_BÁSICAS_DO_PROJETO_1196515.pdf	26/10/2018 15:33:45		Aceito
Projeto Detalhado / Brochura	MP.docx	26/10/2018 15:16:13	TANIA ROBERTA LIMEIRA FELIPE	Aceito

UNESP -FACULDADE DE MEDICINA DE BOTUCATU

Continuação do Parecer: 3.009.068

Investigador	MP.docx	26/10/2018 15:16:13	TANIA ROBERTA LIMEIRA FELIPE	Aceito
Outros	TermoAnuenciagerencia.Pdf	26/10/2018 14:55:34	TANIA ROBERTA LIMEIRA FELIPE	Aceito
Outros	Terminstitucional.pdf	26/10/2018 14:54:59	TANIA ROBERTA LIMEIRA FELIPE	Aceito
TCLE / Termos de Assentimento / Justificativa de Ausência	TCLE.docx	26/10/2018 14:54:03	TANIA ROBERTA LIMEIRA FELIPE	Aceito
Folha de Rosto	FolhaDeRostoAssinada.pdf	26/10/2018 14:53:22	TANIA ROBERTA LIMEIRA FELIPE	Aceito

Situação do Parecer:
Aprovado

Necessita Apreciação da CONEP:
Não

BOTUCATU, 08 de Novembro de 2018

Assinado por:
SILVANA ANDREA MOLINA LIMA
(Coordenador(a))
